# Conception and Concern: A Review of Breast Cancer Risk in Assisted Reproductive Technology

**DOI:** 10.7150/ijbs.105357

**Published:** 2025-03-24

**Authors:** Zijie Guo, Ziyu Zhu, Xixi Lin, Kevin Matthew Zhang, Qingliang Wu, Shenkangle Wang, Mingpeng Luo, Xiaona Lin, Linbo Wang, Jichun Zhou

**Affiliations:** 1 Department of Surgical Oncology, Affiliated Sir Run Shaw Hospital, Zhejiang University School of Medicine, Hangzhou, Zhejiang, 310016, China.; 2 Biomedical Research Center and Key Laboratory of Biotherapy of Zhejiang Province, Hangzhou, Zhejiang, 310016, China.; 3 Loma Linda University School of Medicine, Loma Linda, CA 92350, USA.; 4 The First Affiliated Hospital of Zhejiang Chinese Medical University, Hangzhou, Zhejiang, 310014, China.; 5 The Ninth People's Hospital of Hangzhou, Hangzhou, Zhejiang, 310014, China.; 6 Assisted Reproduction Unit, Department of Obstetrics and Gynecology, Affiliated Sir Run Shaw Hospital, Zhejiang University School of Medicine, Hangzhou, Zhejiang, 310016, China.

**Keywords:** Assisted Reproductive Technology, Breast Cancer, Risk Factors, Reproductive Health, Oncogenic Mechanism, Clinical Research

## Abstract

This review examines the complex relationship between assisted reproductive technology (ART) and the potential risk of breast cancer. Through a thorough analysis, we explore various aspects of this association, considering both the theoretical and mechanistic overlap between ART and breast cancer, as well as the growing body of empirical research aimed at elucidating this relationship. Theoretical analysis suggests that ART exposure inevitably increases levels of reproductive hormones over a relatively short period, potentially elevating susceptibility to breast cancer. However, current clinical evidence does not strongly support this hypothesis, and no direct correlation between ART and breast cancer development has been established. Our study lays the groundwork for informed discussion and offers recommendations for further research in this area of women's health, based on a comprehensive review of both theoretical and clinical research. The findings provide valuable information to guide both specialists and patients in decision-making regarding ART treatment. As we navigate the complexities surrounding conception, this manuscript serves as an essential resource for understanding and addressing the potential risks of breast cancer in the context of ART.

## 1. Introduction

Breast cancer is a prevalent form of cancer worldwide. As indicated by recent statistics on the Global Cancer Burden, in 2020, there were approximately 2.26 million newly diagnosed cases of breast cancer^1^. The occurrence of breast cancer is projected to rise significantly in both economically developed regions and regions experiencing economic transition^2^. Therefore, the prevention of breast cancer is an especially significant global concern in contemporary times. In recent decades, there has been significant advancement in the field of breast cancer epidemiology, leading to the identification of numerous influencing factors associated with the development of breast cancer, including risk and protective factors^3^ (Table 1). There exists a multitude of recognized risk factors associated with breast cancer, which are well documented in numerous studies^4^-^12^. Given that the mammary gland is a hormone-sensitive organ, it is crucial to pay close attention to risk factors for hormone-related breast cancer. It has been documented in the existing literature that elevated levels of endogenous estradiol and progesterone are linked to an augmented susceptibility to breast cancer in both premenopausal and postmenopausal women^13^-^17^. Exposure to exogenous hormones primarily including the use of menopausal hormone replacement therapy (HRT) and oral contraceptives (OC) have also been linked to an elevated risk of breast cancer^18^, ^19^. Conversely, medications with anti-estrogenic properties, such as tamoxifen, have demonstrated efficacy in reducing the risk of breast cancer^20^.

With the rapid advancement of society, women are postponing marriage and the initiation of their first childbirth^21^. Female fertility starts to decrease as early as the age of 30, so postponing childbirth raises the risk of infertility in women^22^. This has resulted in the recognition of infertility as a crucial component of contemporary reproductive medicine. According to an assessment conducted by the World Health Organization (WHO), it has been determined that over 10% of married couples have encountered challenges with infertility^23^. Moreover, it has been estimated that in the year 2020, more than 8 million couples globally sought ART and achieved successful conception^24^. Assisted reproductive technology (ART) has proven to be a valuable solution for women experiencing reduced fertility, successfully fulfilling the fertility goals of numerous families.

However, the administration of assisted reproductive drugs during this process stimulates the production of ovarian hormones, including estrogen and progesterone^25^. The breast is a hormone-sensitive organ, and around 80% of breast cancers are hormone-sensitive. Both estrogen and progesterone, as well as their metabolites, also play important roles in the development and progression of breast cancer^26^. Therefore, the impact of ART on the breast is a matter of significant concern. In the context of clinical implementation of ART, a common concern among most reproductive physicians and patients is whether undergoing ART procedures is associated with an elevated risk of developing breast cancer. This inquiry poses a significant clinical question that necessitates prompt elucidation and response.

## 2. Exploration into ART and hormones pathophysiology in breast cancer

**Figure 1:**
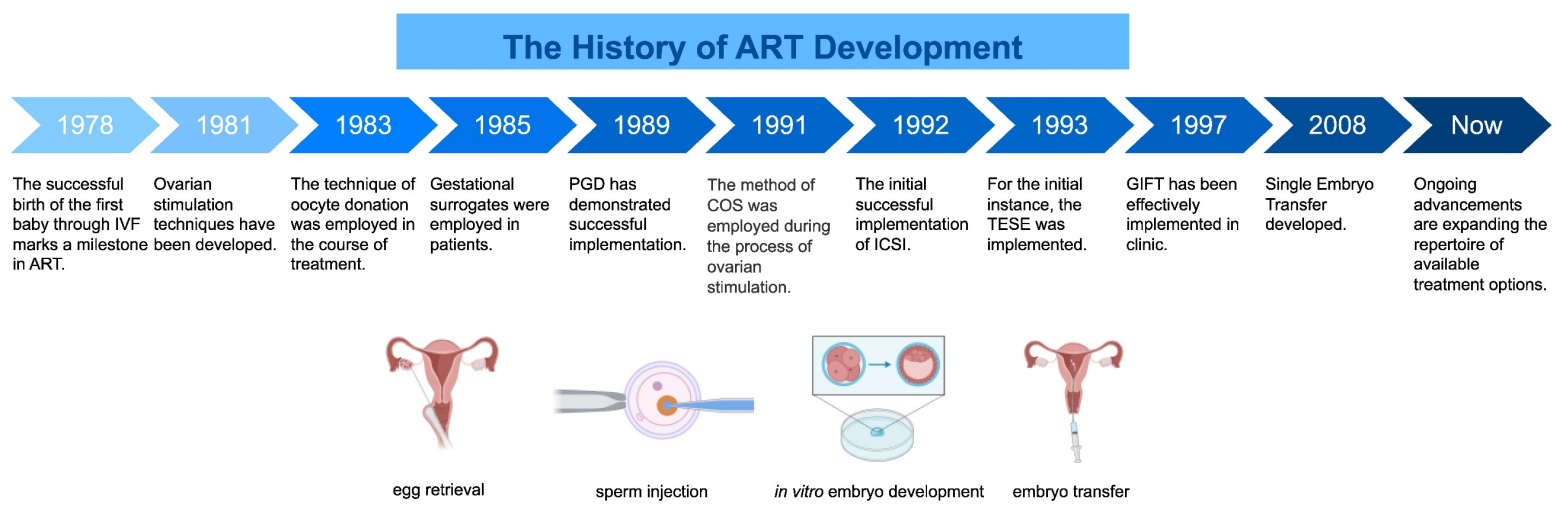
Brief history of ART development. Since its first successful implementation in 1978, ART has been one of the most important technologies in the field of reproductive medicine, and has made great strides over the decades. This figure presents a timeline and offers a brief overview of the historical development of ART therapy, focusing particularly on the invention and application of key technologies. In addition, a brief schematic diagram is included to illustrate some of the techniques commonly used in ART therapy, providing the reader with a clearer understanding of the technology. ART, assisted reproductive technology; IVF, *in vitro* fertilization; PGT, preimplantation genetic testing; COS, controlled superovulation; ICSI, intracytoplasmic single sperm injection; TESE, testicular spermatocyte extraction technique; GIFT, gamete intrafallopian transfer.

Assisted reproductive technology (ART) is a comprehensive clinical concept that encompasses multiple procedures involved in *in vitro* fertilization for reproductive purposes. These procedures include ovarian stimulation therapy (including ovarian stimulation and trigger ovulation), surgical extraction of oocytes from the ovaries, *in vitro* fertilization (IVF) or intracytoplasmic single-sperm injection (ICSI), preimplantation genetic testing (PGT), and embryo transfer (ET)^27^. Several or all of the above operational processes may be included in an ART cycle. Since the initial introduction of IVF in 1978, ART has undergone significant advancements over more than four decades^28^-^32^ (Fig. 1).

Most women who undergo ART typically receive ovarian stimulation therapy. This therapy aims to stimulate the development of follicles and synchronize them to initiate the ovulatory cascade response^33^. Ovarian stimulation therapy involves the use of ovarian stimulants to promote the development and maturation of multiple follicles within a single cycle, ultimately increasing the success rate and number of oocytes available for subsequent fertilization^34^. Numerous ovarian stimulants have been developed and utilized in the ART process. Currently, the most frequently employed ovarian stimulants include gonadotropin-releasing hormone agonists (GnRH-a) and GnRH antagonists^35^, aromatase inhibitors such as letrozole^36^, selective estrogen receptor modulators like clomiphene^37^, human membrane gonadotropin (HMG)^38^ or recombinant follicle-stimulating hormone (FSH)^39^, and human chorionic gonadotropin (HCG)^40^, among others. Controlled ovulation hyper-ovulation (COH), which involves the administration of multiple ovarian stimulating medications, is currently the more prevalent approach^41^. Specifically, in this treatment regimen, a GnRH antagonist is employed to inhibit pituitary function, in order to prevent spontaneous ovulation. GnRH-a is prescribed to stimulate the release of gonadotropins from the pituitary gland and can downregulate by binding to receptors in a supersaturated manner. Gonadotropin is administered to induce ovulation, while progestin is used to counteract the downregulation of GnRH which may impact the luteal phase to prepare the uterus for potential pregnancy and to maintain its functionality, among other considerations^42^, ^43^.

Patients undergoing ART experience a distinct set of physiological changes specific to this treatment modality. Although the changes following a successful embryo transfer are similar to those observed in a natural pregnancy, the process of follicular stimulation are markedly different. In addition to the physiological changes occurring in the body, this process also induces a significant alteration hormone levels within the female body. This hormonal shift is essential for ART, but it has also raised numerous concerns^25^. Through the actions of GnRH and gonadotropins, etc., the levels of HMG, HCG, prolactin, and gonadotropins are elevated during the ART process. Additionally, and most importantly, there is a significant increase in the levels of estrogen and progesterone. The peak circulating estrogen level during an ART cycle is approximately 4,000 pg/mL and can even reach 5500 pg/mL in high cases^44^, ^45^, which is significantly higher than the peak estrogen level of approximately 300 pg/mL during a normal menstrual cycle^46^. Additionally, the peak dose of progesterone exposure during ART cycles is at least twice as high as the peak dose during a normal pregnancy^47^.[48]Estrogen and progesterone are the two main sex hormones in the female body and play crucial roles in a woman's physiological function and health. Nevertheless, the elevated hormone stimulation associated with reproduction also gives rise to concerns, particularly in light of the potential risk of breast cancer, a disease intricately connected to hormone. According to a substantial amount of research, estrogen, and progesterone have been found to exert not only independent effects, but their metabolites also appear to play a significant, if not more crucial and definitive, role in the development and progression of breast carcinoma. Estrogen is primarily synthesized in the female body by the ovaries. Figure S1 illustrates the pathway by which estrogen is synthesized in the body^49^, ^50^. Then the metabolism of estradiol and estrone results in the formation of catechol-estrogens through three irreversible competitive pathways, including the production of 2-hydroxyestrone (2-OHE1), 4-hydroxyestradiol (4-OHE2), estriol, etc.^51^ Catechol estrogens are not stable metabolite forms and are subsequently metabolized by oxidation and conjugation^52^ (Figure S2). In the female body, it has been observed that estrogen levels in breast tissue are significantly higher, ranging from 10 to 50 times higher than blood levels^53^. Furthermore, studies have detected the presence of estrogen metabolite and conjugate levels ranging from 3-13 pmol/g in female breast tissue, indicating the active involvement of the estrogen metabolic pathway in human breast tissue^54^. On the other hand, progesterone, similar to estrogen, is highly susceptible to over 100 progesterone metabolites through redox processes^55^. Like estrogen, progesterone and its metabolites accumulate in significant amounts in the mammary gland in an active form^56^. Thus, the breast is an organ that is characterized by, and strongly influenced by, elevated concentrations of estrogen, progesterone, and their metabolites.

Breast carcinoma is a malignant tumor that result in uncontrolled multiplication of abnormal cells with the potential to invade other parts of the organism. The process of its carcinogenesis is typically regarded as a multi-hit sequence that starts with initiation at the genetic level and culminates in promotion and enhanced proliferation^57^. As previously stated, the mammary gland, a hormone-sensitive organ, exhibits a strong correlation between hormone levels and the development of cancer. Particularly, hormones associated with reproductive processes in women, such as estrogen and progesterone, play a significant role in the carcinogenic process of the mammary gland. It is important to note that estrogen and progesterone primarily affect hormone receptor-positive breast cancers, while having less of an effect on triple-negative breast cancers, and the incidence of triple-negative breast cancers is at the lower end of the spectrum of breast cancers^58^. Therefore, in the literature or studies discussing the relationship between estrogen and breast cancer, the breast cancers studied are generally considered to be hormone receptor-positive breast cancers. The concentration of estrogen is found to be higher in malignant breast tissue compared to non-malignant tissue^53^, and many existing reports in the literature have demonstrated elevated levels of estrogen in the blood are associated with an increased risk of developing breast cancer^59^. Regarding progesterone, the limited availability of accurate tests and low levels of circulating progesterone have hindered attempts to conduct epidemiologically relevant studies on the association between endogenous progesterone levels and breast cancer. However, it has been observed that long-term exposure to high doses of exogenous progesterone, in combination with estrogen, from sources such as the use of short-acting contraceptives or postmenopausal hormone therapy can increase the risk of breast cancer^18^, ^60^. Additionally, a mounting body of research indicates a significant correlation between endogenous progesterone levels and the risk of developing breast cancer^61^. Pike *et al.* conducted a study that revealed that the risk of breast cancer could potentially be influenced by the cumulative exposure of breast tissue to estrogen and progesterone^62^. This suggests that the development of breast carcinoma may be attributed to a combination of multiple factors, or "multiple strikes".

## 3. The impact of ART on breast cancer - theoretical perspectives

In this section, we will conduct an analysis from a theoretical perspective based on principles from molecular biology, cell biology, histology, and related mechanisms in order to analyze how changes in hormone levels induced by ART, particularly estrogen and progesterone, may impact the sites and mechanisms involved in the development of breast cancer.

### 3.1 Oncogenic estrogen and progesterone signaling

**Figure 2:**
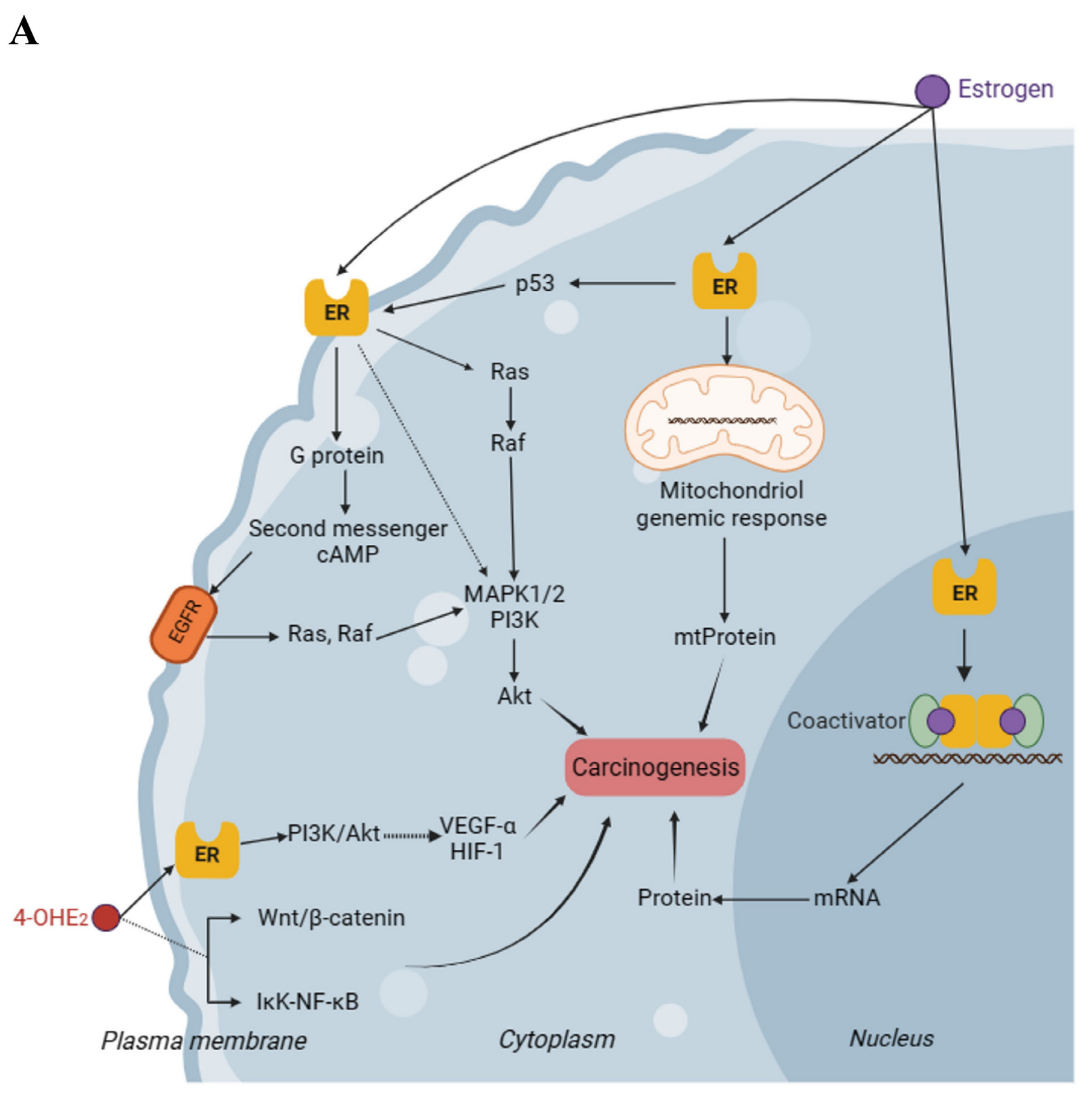

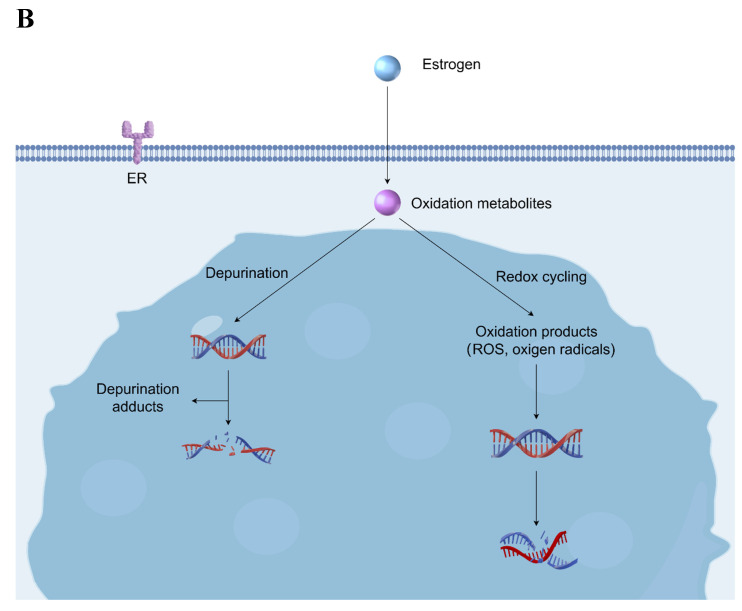
Carcinogenic mechanisms of estrogen and its metabolites in breast cells. **A.** This figure illustrates the potential oncogenic signaling pathways of estrogen and its metabolites in breast cells. There are three main oncogenic signaling pathways associated with estrogen, all of which involve the participation of ER. 4-OHE2, an estrogen metabolite, also plays a significant role in the oncogenic signaling pathway. It may act on ER to induce oncogenic signals and may also be capable of generating oncogenic signals through a pathway that is independent of ER. **B.** This figure illustrates the mechanism through which the genotoxicity of estrogen and its metabolites leads to breast cancer. The potential mechanisms primarily involve depurination and redox cycling. This genotoxic effect ultimately leads to damage or breakage of the DNA structure, thereby causing the transformation of breast cells into cancerous cells. cAMP, cyclic adenosine monophosphate; 4-OHE2, 4-hydroxyestradiol; ER, estrogen receptor; EGFR, epidermal growth factor receptor; mtProtein, mitochondrial protein; mRNA, messenger RNA; MAPK, mitogen-activated protein kinase; PI3K, phosphoinositide 3 kinase; HIF-1, hypoxia-inducible factor-1; VEGF, vascular endothelial growth factor; NF-ĸB, nuclear factor-kappa B; ROS, reactive oxygen species. Dashed-line arrows indicate putative pathways.

The interaction of estrogen and progesterone with their cognate receptors and subsequent activation of various signaling pathways is recognized as a significant factor in the initiation and progression of breast carcinogenesis. This theoretical mechanism serves as a convincing link between ART treatment and a potential elevation in the risk of breast cancer. Numerous studies have documented the involvement of estrogen-related signaling pathways in the pathogenesis of breast carcinoma from various angles, and relevant mechanisms have been fully described^51^. Activation of estrogen receptor (ER) by estrogen triggers various signaling pathways, resulting in transcription factors that facilitate the progression of cancer. Among various signaling pathways implicated in breast cancer, the Ras/Raf/MAPK pathway has been extensively investigated. It has been found that breast cancers in patients with overexpression of Ras and MAPK proteins exhibit a more aggressive phenotype^63^. Estrogen can activate various protein kinases, including the original activated protein kinase known as silk crack, and this activation leads to an enhancement of second messenger systems, such as cyclic adenosine monophosphate (cAMP) levels. These mechanisms are crucial in regulating cell proliferation and inhibiting apoptosis^64^, ^65^. Also, it has been discovered that the transcriptional regulation, facilitated by estrogen-activated ERα and p53, results in the suppression of ERβ expression in breast cells^66^, while ERβ has been demonstrated to have a protective effect against breast tumorigenesis^67^. The signaling pathways related to estrogen in breast carcinogenesis have been extensively studied and documented for many years, thus we provide a comprehensive schematic (Fig. 2A) representation without delving into excessive detail here.

Additionally, endogenous levels of progesterone are significantly increased during ART, and they may also have a contributing effect on the development of breast cancer through associated signaling pathways. In a comparative analysis of tissues obtained from the follicular and luteal phases, it was observed that the expression of 221 genes was significantly upregulated during the luteal phase. These genes are associated with various pathways related to the cell cycle, mitosis, and DNA damage and repair. Additionally, abnormal expression of three paracrine factors, namely RANKL, WNT4, and ectodomain proteins was detected^68^. These findings suggest a potential correlation between elevated levels of endogenous progesterone and the development of breast cancer. Researchers have also found that progesterone can directly regulate the microenvironment of breast organogenesis and breast tumors through the Notch signaling pathway, and regulate the self-renewal and differentiation of breast stem cells, thereby activating the signal for breast proliferation. Interestingly, this process has been found to contribute to the development of more aggressive forms of breast carcinoma^69^, ^70^.

### 3.2 Atypical ER and PR expression

Previous research has also indicated that changes in estrogen and progesterone levels in human can directly influence the expression of ER and PR, thereby affecting breast cancer development. Graham *et al.* have comprehensively summarized the available evidence on this topic that normal breast tissue and primary cell models have demonstrated that endogenous high levels of estrogen in normal human breast tissue result in increased expression of ER and PR^69^. It is widely accepted that receptor-positive cells do not directly respond to hormonal signals, but instead promote the proliferation of breast cells through paracrine effects on surrounding receptor-negative cells^71^. Moreover, in hormone receptor-positive breast cancer cells, there is an increasing number of aberrantly proliferating cells expressing steroid hormone receptors^72^. These steroid receptor-positive cells undergo a switch to autocrine signaling mechanisms, which is not unrelated to high levels of steroid hormones^73^, ^74^. Therefore, exposure to high-dose estrogen and progesterone through ART leads to an increase in ER and PR expression in breast cells, and it is possible that some still-unknown mechanism may cause this fraction of steroid receptor-positive cells to transition from a paracrine to an autocrine signaling mechanism, thereby inducing breast cancer development and progression.

Additionally, PR can be classified into two isoforms, namely PRA and PRB. In normal mammary epithelium, both isoforms are typically expressed in equal amounts^75^. However, inappropriate exposure to exogenous progesterone or its analogs can lead to abnormal expansion of progenitor or progenitor-like cells in the human mammary gland^73^, which can disrupt the balance between PRA and PRB in all PR-expressing mammary cells. Studies have shown that this imbalance between PRA and PRB ratios occurs early in the development of breast cancer, and the isomer ratio gradually increases with the progression of breast cancer^76^. More importantly, the alteration in the expression ratio of PRA and PRB may have significant implications for breast cancer progression observation and treatment options. It could serve as a monitoring indicator for early detection of breast cancer and the implementation of personalized treatment regimens, offering promising prospects for breast cancer patients.

### 3.3 Breast proliferation and involution

It is also worth noting that the accelerated development of mammary glands, induced by high doses of estrogen and progesterone, may also serve as an alternate explanation for the potential elevated susceptibility to breast cancer associated with ART. The process of mammary gland development and maturation is a complex and intricate process that is regulated by systemic hormones and local growth factors. Throughout this process, the mammary gland's environment, structure, and cells are constantly changing^77^. This indicates that the developmental maturation of the mammary gland is heavily influenced by the endocrine environment^78^. Recent research has confirmed that estrogen plays a crucial role in regulating ductal elongation during the development of the mammary gland^79^, while progesterone is responsible for regulating the development of mammary collateral and lobular structures^80^. When breast tissue is exposed to elevated levels of estrogen and progesterone during the ART process, it triggers a process of mammary gland development that closely resembles that of pregnancy. This process involves the expression of signals that form luminal alveolar cells and tertiary ductal and lobular alveolar structures, facilitating the expansion of ductal tissue and differentiation of mammary epithelial cells^77^. The density of mammary lobules increases during this developmental process, which is believed to be the primary source of cancer precursors. Studies conducted on women who have undergone benign breast biopsy have demonstrated that a decrease in the level of lobular regression is associated with an elevated risk of breast cancer. In other words, the development of the mammary glands, influenced by estrogen and progesterone, leads to an increase in the density of the lobules, which is considered a potential risk factor for breast cancer^81^, ^82^.

The development and maturation of the mammary gland exhibits a complex and dynamic process, rather than a simple and stable progression. After a transient exposure to high doses of reproductive-related hormones, and in the absence of sustained stimulation, the mammary gland also experiences a process of remodeling referred to as degeneration. During this process, the structural configuration of the gland gradually returns to its pre-hormone exposure state. During the degenerative process, the mammary gland undergoes not only apoptosis of structures such as alveoli and epithelium but also remodeling of fat, extracellular matrix (ECM), and changes in the immune microenvironment^77^. The ECM undergoes a significant reconstruction of fibrillar collagen during this process, which does not affect the ongoing formation of fat. This change can mediate alterations in the protective effects against cancer^83^. High fibrillar collagen in the ECM, along with a high mammographic density, is associated with a more than four-fold increase in the risk of cancer^84^. Furthermore, the recruitment and activation of immune cells during involution can potentially modify the immune microenvironment of the body, leading to the formation of a tumor microenvironment and an increased risk of subsequent breast cancer^85^-^87^. If ART treatment is not completed in one session, the mammary gland will experience an involution after transient high-dose exposure to reproduction-related hormones. The idea that the involution process, not just the glandular development process, increases the risk of breast cancer development not only helps to explains how a single ART session can increase cancer risk, but also provides a theoretical explanation for the association between multiple ART sessions and a higher risk of breast cancer.

On the cellular level, the proliferation that is associated with mammary gland development and maturation holds the potential to increase the risk of breast cancer as well. In the mammary gland, estrogen and progesterone induce the expression of cyclin D1. Cyclin D1-dependent mechanisms can promote tumor invasiveness and high proliferative activity^88^. This mechanism has been found to play a significant role in the carcinogenesis of breast cells and the progression of cancer cells^89^. Paracrine secretion is recognized as an important mechanism through which estrogen and progesterone contribute to the development of the mammary gland. Activation of multiple paracrine signaling pathways, such as up-regulation of amphiregulin and activation of mediators like WNT4 and NF-κB ligand (RANKL), by estrogen and progesterone, can lead to the extensive proliferation of breast cells. It is still worth noting that these processes are also associated with the carcinogenesis of breast cells^90^, ^91^. Furthermore, progesterone can exhibit exclusive mechanisms in mammary gland development, making it the most crucial proliferative hormone in this context. The follicular phase, characterized by higher serum progesterone levels than estradiol levels, exhibits lower levels of mitosis and proliferation compared to other phases^92^. Progesterone, known for its potential mitogenic activity, possesses a unique mechanism whereby it stimulates progenitor cell proliferation in the normal mammary gland and plays a role in tissue-specific responses^93^. The effects of progesterone-induced progenitor proliferation not only promote the development of the mammary gland by increasing the proliferation of normal luminal progenitors but also contribute to the expansion of cancer-sensitive luminal progenitor populations, thereby influencing the development of breast cancer. Additionally, researchers have discovered that telomere dysfunction may occur in normal luminal progenitors during this rapid proliferation process^94^, and meanwhile, extensive telomere fusion has been observed in early breast cancer lesions^95^, providing potential evidence for the theory of the specific cellular origin of breast cancer. Thus, the administration of ART treatment, which results in heightened exposure to estrogen-pregnant stimuli, not only facilitates the regular proliferation and differentiation of breast cells but also potentially fosters cellular carcinogenesis and the advancement of malignant cells.

### 3.4 Metabolite genotoxicity

Numerous studies have indicated that, in addition to the direct participation in the oncogenic signaling pathway, estrogen and progesterone metabolites, particularly catechol estrogens, may also play a role in this process through distinct mechanisms. It has been observed that 4-OHE2 exhibited a greater capacity to stimulate the proliferation of breast cancer cells at lower concentrations when compared to other estrogen-related compounds^96^, ^97^. On the other hand, 4-OHE2 not only induces cellular transformation pathways but also enhances the growth of cancer cells by activating specific intracellular signaling^98^, ^99^. Exposure to high doses of estrogen from ART, it is evident that the production of 4-OHE2 also experiences a significant increase. This phenomenon also plays a crucial role in the initiation of breast cancer.

Lareef *et al.* conducted an experiment where they treated mammary epithelial cells with E2 and its catechol metabolites. They discovered that even in the presence of anti-estrogen treatment, these metabolites could induce DNA damage and tumorigenic transformation of the cells *in vitro*, suggesting that the genotoxicity of estrogen metabolites may play a role in the process of cellular transformation^100^. The genotoxicity primarily results from the DNA damage caused by estrogen metabolites, leading to genetic mutations and these mutations can accumulate over an extended period, ultimately inducing neoplastic transformation^101^. Recent studies have identified multiple potential mechanisms associated with this process (Fig. 2B). The first notable aspect is that certain metabolites of estrogen induce the release of estrogen-adenine/guanine adducts from DNA *via* a depurination mechanism, resulting in structural impairments to the DNA^102^. Another reason is that substances such as quinones and semiquinones produced by the oxidation of catechol estrogen metabolites and catechol estrogens activated by lactoperoxidase can undergo redox cycling during metabolism, producing oxidation products that can lead to DNA damage and mutations^103^. The theoretical implication is that a patient undergoes ART, there is a temporary and significant elevation in endogenous estrogen levels within the body. In women's bodies, when an imbalance in estrogen metabolism occurs, characterized by elevated levels of endogenous or exogenous estrogen, there is an overexpression of estrogen-activating enzymes and a deficiency of inactivating enzymes. Consequently, the levels of estrogen quinone and depurin adducts are elevated, leading to more severe DNA damage^100^. Furthermore, it is believed that an elevated dosage or additional exposure to estrogen can result in an increase in oxidative free radicals within breast tissue. Fussel *et al.* conducted a study that revealed that treatment with catechol estrogen led to the generation of higher levels of hydroxyl radicals and H_2_O_2_ in the lysates of mammary epithelial cells, ultimately resulting in oxidative modification of DNA^104^.

Unlike with estrogen, there is a scarcity of studies that provide substantial evidence for the involvement of progesterone in the promotion of breast carcinogenesis through genotoxicity. No evidence of DNA damage induced by progesterone has been observed in the *in vivo* or *in vitro* experiments^105^, ^106^. Furthermore, no direct DNA damage was detected through the measurement of DNA adducts in the livers of patients undergoing mono-progesterone treatment^107^. Therefore, it should not be assumed that exposure to high-dose progesterone through ART leads to breast carcinoma *via* genotoxic DNA damage specifically.

### 3.5 Oncogenic metabolite signaling

In addition to causing genotoxic DNA damage, catechol estrogen metabolites are themselves capable of participating in oncogenic signaling pathways, especially 4-OHE2. In the context of breast carcinogenesis, the mechanism of 4-OHE2 appears to involve two distinct pathways: ER-dependent and non-ER-dependent (Also shown in Fig. 2A). The ER-dependent pathway is primarily associated with the PI3K-AKT signaling pathway. In the investigation of the malignant transformation of MCF10A cells, it was observed that the activation of phosphatidylinositol 3-kinase (PI3K) occurs after the treatment of cells with 4-OHE2. Additionally, it was found that PI3K may further induce an elevation in the phosphorylation of serine/threonine protein kinase (Akt). This increase in phosphorylation is believed to mediate the cancerous process in breast cells^96^. Meanwhile, previous studies have provided evidence that 4-OHE2 can stimulate the expression of hypoxia-inducible factor-1 (HIF-1) and vascular endothelial growth factor A (VEGF-A) through the PI3K/AKT signaling pathway in a specific human ovarian cancer cell line^108^, ^109^. Furthermore, non-ER-dependent pathways also seem to contribute to the development of breast carcinoma through the action of 4-OHE2. Kwon *et al.* demonstrated that the enzyme CYP1B1 plays a role in promoting cancer cell survival by upregulating the specificity protein 1 (Sp1), which is involved in DNA hypermethylation-mediated inhibition of death receptor 4 (DR4). Additionally, CYP1B1 may induce epithelial-mesenchymal transition (EMT) and activate the Wnt/β-catenin signaling pathway, both of which contribute to the progression of the carcinoma. Interestingly, it was also observed that treatment of MCF cells with 4-OHE2 produces similar effects to CYP1B1 overexpression^110^, ^111^. This finding suggests a potential new mechanism for 4-OHE2-induced tumorigenesis. Furthermore, not only elevated levels of ROS produced during the metabolism of estrogen enhance the DNA binding capacity of nuclear factor-kappa B (NF-ĸB) by stimulating the activity of Ikappa B kinases alpha and beta, but also the exposure to 4-OHE2 result in the transient activation of NF-ĸB. That is to say, estrogen and its metabolite 4-OHE2 have the potential to exert an influence on the development of breast cancer cells by modulating the IĸK-NF-ĸB signaling pathway^112^, ^113^. As for 16α-OHE1, it is found that its concentration in breast cancer tissues is eight times higher compared to nearby mammary adipose tissues^114^, and additionally, animal experiments have indicated that 16α-OHE1 may stimulate extra-programmed DNA synthesis in mammary epithelial cells by promoting the expression of the Ras oncogene, thus potentially contributing to breast carcinogenesis^115^.

### 3.6 Other hormone signaling

In addition to short-term fluctuations in estrogen and progesterone during ART, elevated levels of prolactin (PRL), HCG, gonadotropins, and other hormones may also affect the development of breast cancer. PRL is a hormone that interacts with its respective receptors and progesterone in women, leading to the proliferation of mammary ductal and luminal epithelial cells^116^. Both *in vitro* experiments and epidemiological studies have demonstrated a stimulatory effect of PRL on the growth of human breast cancer cells^117^, ^118^. This may be attributed to PRL's ability to influence feedback from the RANKL pathway and subsequently affect epithelial cell processes through mammary luminal progenitors^116^. Additionally, PRL may regulate the actin cytoskeleton *via* the Src pathway, thereby promoting cancer cell proliferation, a significant factor in the development of cancer^119^. HCG is also an important hormone associated with pregnancy and can be used as an ovarian stimulator during ART, leading to a rapid increase in HCG levels in women's bodies. The impact of HCG on cancer development is currently a subject of controversy. Some studies suggest that HCG can reduce the carcinogenesis of breast cells and exhibit anti-proliferative effects on breast cancer cells by down-regulating estrogen receptors and reducing the susceptibility of breast tissue to toxic substances^120^, ^121^. However, it appears that this anticancer effect is limited to placental HCG, while β-HCG seems to have a tumor-promoting function and is associated with a poor prognosis of breast cancer^122^.

## 4. The impact of ART on breast cancer - a systematic review of clinical studies

### 4.1 Methods of systematic review

From aforementioned theoretical standpoint, the administration of ART treatment is expected to result in elevated levels of estrogen, progesterone, and other reproductive hormones, as well as hormone metabolites, within the patient's body. Consequently, this phenomenon may potentially serve as a risk factor for the onset and progression of breast cancer. Nevertheless, given the intricate nature of the ART procedure, there is currently no foundational research available that explicitly substantiates the claim that ART can result in a heightened susceptibility to breast cancer. Besides, after undergoing ART, patients may subsequently engage in childbirth, breastfeeding, and other physiological processes that have been shown to provide protective effects against the development of breast cancer. Therefore, in order to establish the clinical relevance of previous theoretical analyses, it is imperative to conduct a comprehensive review and analysis of pertinent clinical studies^123^.

In this section of the article, a comprehensive systematic review will be conducted on the notable clinical studies published within the last two decades regarding the correlation between ART and the risk of breast cancer. We try to elucidate any potential association between ART and susceptibility to breast cancer. To identify pertinent clinical studies, an extensive search was initially conducted utilizing databases such as PubMed, Web of Science, and other relevant sources. We conducted our study by examining articles that were published in the past two decades (from January 2003 to December 2023). Our search scheme table is presented in the table in Supplementary information 2. All articles that were retrieved were manually downloaded in their entirety. The data was subsequently integrated into a database utilizing EndNote X9 to ensure consistent management. The studies examined in this section of the review exclusively encompassed case-control, retrospective, and prospective cohort studies. All articles included in the study met the following inclusion criteria: they had complete full text available, were written in English, and had complete keywords in the title or abstract^124^. We have also registered related protocols on Prospero under the code CRD42023494618.

**Figure 3:**
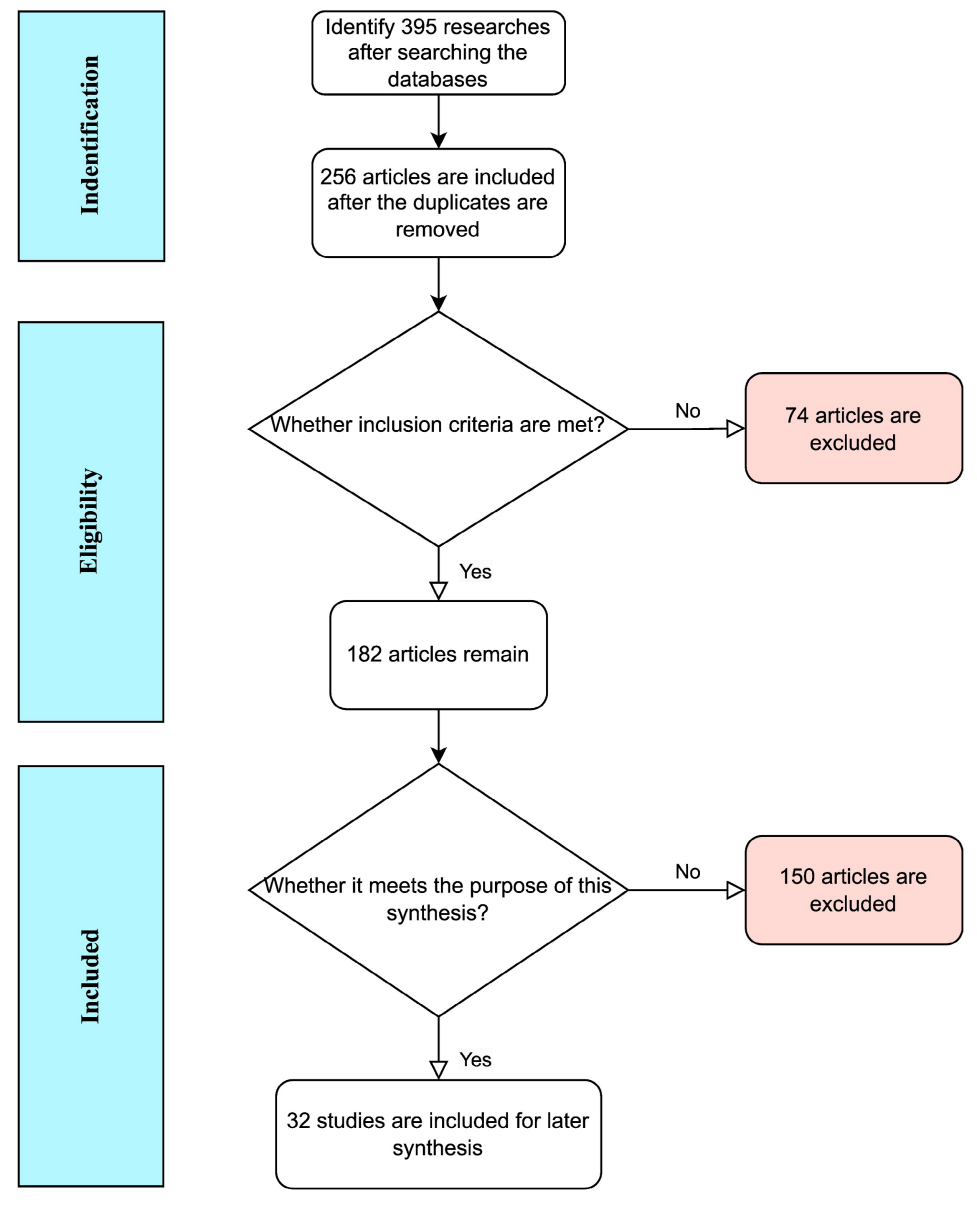
Flowchart of the included eligible studies. This is a schematic representation of endogenous estrogen metabolism. In the female body, estradiol and estrone are metabolized into catechol estrogens through several competitive and irreversible pathways. Catechol estrogens are intermediate products of metabolism. They are not very stable and can undergo further metabolism catalyzed by different enzymes to produce more stable products for excretion or storage or to produce active products that cause other biochemical reactions in the cells. E1, estrone; E2, estradiol; CYP, cytochrome P450; 2-OHE1, 2-hydroxy estrone; 4-OHE1, 4-hydroxy estrone; 2-OHE2, 2-hydroxy estradiol; 4-OHE2, 4-hydroxy estradiol; 2-MeOE2, 2-methoxy estradiol; 4-MeOE2, 4-methoxy estradiol; 2-MeOE1, 2-methoxy estrone; 4-MeOE1, 4-methoxy estrone; COMT, catechol-O-methyltransferase; Q, quinone; SQ, semiquinone; 6-N3-Ade, 6-N3-Adenine; 1-N7-Gua, 1-N7-Guanine.

Based on the aforementioned criteria, a comprehensive search of the selected databases was conducted, resulting in the inclusion and review of a total of 395 articles in the preliminary study. Fig. 3 illustrates the flow of this inclusion of articles. A total of 32 articles meet the predetermined purpose and inclusion criteria, and were deemed eligible for inclusion in this review of clinical studies.

In addition to conducting the standard data abstraction and analysis of the articles included in our study on the association between ART and breast cancer risk, we also evaluated the potential errors present in these clinical studies (Table S1). To facilitate a more thorough examination of the issues and potential sources of errors in these clinical studies, we systematically classified the potential sources of errors according to the experimental design, experimental procedure, and data analysis. Additionally, codes were allocated to signify the specific type of errors^125^. For this review, all articles were thoroughly examined and categorized according to the potential errors previously discussed. Through conducting these analyses, our objective is to enhance the evaluation of the reliability and validity of the findings and interpretations. We also aim to elucidate the reasons behind the divergence of certain clinical studies from the conclusions drawn in previous theoretical analyses. The progressing objective is to offer valuable insights aimed at enhancing the design of clinical experiments and addressing current challenges.

It is crucial to emphasize that, apart from the significance of acquiring precise and comprehensive data regarding variables related to the study, such as the treatment regimen and duration, the influence of confounding factors on the study outcomes should not be disregarded, since they can significantly impact the results. These factors are recognized or assumed to be risk factors for breast cancer and failure to exclude or adjust for these factors in different groups can lead to an imbalance and subsequently affect the study results. This, in turn, can influence the assessment of the risk level associated with breast cancer or potentially alter the study's conclusions. There exists a multitude of confounding factors, and the ones presented in Table 1 exert a significant influence on the susceptibility to breast cancer. It is evidently challenging to account for all the confounding variables in a study involving patients undergoing ART or in the broader population. Therefore, this paper aims to examine the impact of age, fertility (including number of births and time to first birth), family history of breast cancer, and other hormonal treatments (such as oral contraceptives and hormone replacement therapy) on the risk of breast cancer in patients undergoing ART. This analysis is conducted in conjunction with the consideration of confounding factors that have been highlighted in clinical studies and theoretical analyses, as these factors may have a significant and discernible influence on breast cancer risk in ART patients. The association between pregnancy, fertility-related factors, and the risk of breast cancer has been extensively studied and documented in various scholarly articles^77^, ^126^, ^127^. Also, it is evident that the administration of other hormonal therapies to the patient can exert a substantial influence on the susceptibility to hormone-sensitive breast cancer^18^, ^19^. Regarding the influence of family history as a confounding factor, it is widely accepted that family history is associated with an elevated risk of developing various types of cancers. Gauthier *et al.* have proposed in their article that there might be a significant correlation between family history and the utilization of ovulation-promoting drugs in the onset of breast cancer^128^.

### 4.2 Results and analysis of the systematic review

There are 32 articles on clinical studies that were included in the review analysis. Detailed information about the study is presented in Table S2. Among these articles, 7 concluded that there was an overall increase in the risk of subsequent breast cancer in patients treated with ART^33^, ^125^, ^129^-^133^. Another 8 articles did not conclude that ART treatment as a whole led to an increase in the risk of breast cancer but rather found an association between ART treatment and the development of breast cancer in specific populations, specific treatment regimens, or certain types of breast cancer^44^, ^123^, ^134^-^139^. One article provided indirect evidence that ART causes an increased risk of breast cancer^140^. However, the findings of 7 other articles suggest that ART treatment may reduce a patient's risk of developing breast cancer^141^-^147^. The remaining 9 articles did not find a clear correlation between ART and the risk of developing breast cancer^128^, ^148^-^155^.

There were eight studies that, while failing to find a relationship between ART and overall breast cancer risk, found an association between ART and breast cancer development under specific conditions^44^, ^123^, ^134^-^139^. This deserves more attention and analysis with a view to discovering more relevant information. A comprehensive cohort study conducted in Great Britain examined a large population and reported their findings. The study revealed that there was no significant alteration in the overall risk of breast cancer (SIR = 0.98, 0.94-1.01) among the participants. However, a slight elevation in the risk of *in situ* breast cancer (SIR = 1.15, 1.02-1.29) was observed in individuals who underwent ART^139^. In a cohort study conducted, there was no overall increase in the proportion of women treated with IVF who developed breast cancer (HR = 1.10, 95% CI 0.88-1.36), while there was an increase in the proportion of women who started IVF at a young age (under 24 years) and developed breast cancer (HR = 1.59, 95% CI 1.05-2.42)^44^. This finding conflicts with the findings of Vassard *et al.*^33^,and a comparative analysis of the two studies may yield interesting conclusions. Lerner-Geva *et al.* discovered that women treated with clomiphene citrate had a significantly higher risk of breast cancer (SIR = 1.4; 95% CI 1.0-1.8)^135^. Also, Reigstad *et al.* indicate that there was an increased risk of breast cancer in women who had successfully given birth (HR = 1.26; 95% CI 1.03-1.54)^138^. Burkman *et al.* also found that the relative risk of breast cancer was significantly higher for women using hMG for an extended period (OR = 2.7, 95% CI 1.0-6.9)^137^. Kristiansson *et al.* found an increased risk of breast cancer with progesterone use during ART (RR = 3.36; 95% CI 1.3-8.6)^134^. The details of these studies, the conclusions of the results, and the presence of errors can also be obtained in Table 2A-B.

It has become a consensus among scholars that pregnancy and lactation are protective factors against breast cancer. Pregnancy and breastfeeding are common processes that occur during a woman's reproductive life. These processes protect against the development of breast cancer through various mechanisms, including reduced estrogen exposure, enhanced mammary cell differentiation, regular emptying of the mammary gland, and a reduced risk of inflammation^16^, ^156^. For infertile women, who are inherently at higher risk for breast cancer than the general population due to infertility, ART has enabled many infertile women to become pregnant, while some patients remain infertile after ART^77^. This situation has raised questions among physicians and patients about whether fertility status after ART also affects the risk of breast cancer. Among the clinical studies we reviewed, there were 5 studies related to this topic^33^, ^136^, ^138^, ^150^, ^154^. However, upon analysis, we found that the results of these studies were not consistent. Therefore, based on the current clinical studies, it is challenging to determine whether there is a definitive association between pregnancy and breastfeeding after ART treatment and the subsequent risk of breast cancer. This issue requires further clarification through additional clinical studies.

Interestingly, it has been observed that mutations in the BRCA gene have a significant influence on both infertility and the risk of developing breast cancer. There is a consensus among researchers that mutations in the BRCA1/2 gene can lead to a substantial elevation in the risk of breast cancer. A growing body of evidence suggests that BRCA gene mutations can potentially have detrimental effects on ovarian reserve function in women and may lead to infertility^157^, ^158^.This phenomenon gives rise to further concerns among clinicians and genetically susceptible women who are considering ART treatment, as they are apprehensive about the potential increased risk of breast cancer following the treatment^159^.A total of three clinical studies that were relevant to this topic were included in our analysis. A large cohort study conducted by Machtinger *et al.* indicated that there was no significant difference between the group receiving ART and the general population in terms of the proportion of women with BRCA1/2 mutations among those diagnosed with breast cancer^148^. Similar correlations were reported by Derks-Smeets *et al.* and Perri *et al.* in their studies^146^, ^147^. Nonetheless, the current results do not directly alleviate the concerns of the patients involved, as the sample sizes of the current studies are still small. Hence, there exists a challenge in differentiating the population with BRCA mutations from the general population to draw personalized conclusions regarding the correlation between ART and the risk of breast cancer. Additional clinical investigations in this domain are imperative and hold significant clinical relevance.

According to the analysis of the significant clinical studies that were incorporated in this review, the conclusions derived from these studies were not uniform and were, in fact, contradictory. Based on the findings of the analysis, it is inconclusive to determine a definitive correlation between ART and the risk of breast cancer, whether positive or negative. The results suggest that there is no precise association between ART and the risk of breast cancer in patients, which aligns with the conclusions drawn in previous reviews^160^, ^161^ and meta-analyses^25^, ^162^-^164^. Additionally, as previously stated, each of the clinical studies included in our analysis exhibited various factors that may have introduced errors into the experimental results. These factors could be attributed to different conditions or unavoidable issues encountered during the studies. Hence, it is crucial to approach the analysis and interpretation of these findings with prudence and critical evaluation. Since these findings are ambiguous and subject to scrutiny, more comprehensive and standardized clinical studies are still required to address this issue.

## 5. Prospects for research and practices

In the aforementioned theoretical analysis, various viewpoints have been investigated, indicating that the significant increase in estrogen, progesterone, HCG, prolactin, and other hormones and hormone derivatives resulting from assisted reproductive technology (ART) may potentially contribute to the initiation and progression of breast cancer. This assertion aligns with the prevailing consensus among numerous clinical practitioners and serves as a significant cause for apprehension among both medical professionals and patients. However, our subsequent review and analysis of recent clinically significant studies have resulted in disparate findings. Our analysis indicates a dearth of substantial evidence to establish a direct correlation between ART treatment and the risk of breast cancer development in patients. Nevertheless, it is crucial to acknowledge that the potential association between ART and breast cancer, while still uncertain and a topic of ongoing debate, does not pose a significant enough risk to compromise the breast health of individuals undergoing this particular treatment modality. This discovery holds the potential to address the concerns of patients and healthcare providers regarding the potential cancer risk linked to ART.

In the clinical context, it is frequently observed that infertile women undergoing ART treatment often express concerns about potential side effects, including the development of breast cancer. However, the pronounced and conspicuous desire of these women to conceive and bear children surpasses these concerns, thereby rendering their motivation more conspicuous and persuasive^165^. As for the risk associated with ART, it is worth noting that although breast cancer is a prevalent form of cancer with a rising incidence rate, the overall risk to the population remains low, given that the incidence of breast cancer ranges from 30 to 90 cases per million individuals worldwide.^2^, ^166^. In fact, based on the existing clinical research data, it has been observed that in studies examining the association between ART and breast cancer risk, the relative risk (RR) identified is often below 1.5, indicating a relatively low increase in risk. Overall the advantages of ART are much greater than any potential disadvantages. Therefore, healthcare providers should not have concerns about refraining from offering ART to patients due to potential risks of breast cancer.

Along with economic advancement and increased accessibility of information to the public, there is a rising public apprehension regarding the efficacy of medications and treatment programs. However, this research interest is not only exclusively centered on the effectiveness of the intervention but also encompasses an examination of the potential adverse effects and long-term implications. Furthermore, there has been a discernible increase in women's engagement with breast health, a phenomenon that carries substantial implications, as expounded upon in this scholarly article. When the potential risks associated with ART and breast cancer are not adequately and concisely explained to the patient, it may result in difficulties for the patient in making informed decisions about their treatment and result in unnecessary anxiety^167^, ^168^. Therefore, clinicians and researchers must undertake additional investigations into the association between ART and the potential risk of breast cancer, in order to elucidate the presence and extent of the risk. Thus, it remains crucial to undertake further rigorous and refined clinical studies. To advance our comprehension of the correlation between susceptibility to breast cancer and its association, we have compiled a set of recommendations on the most effective strategy for designing and implementing meticulously planned clinical trials and fundamental experiments, while also introducing innovative concepts into study design (Table 3).

The distinctive attributes of ART treatment present notable medical and ethical complexities, rendering it unfeasible to carry out RCTs. Consequently, it is imperative to consider alternative study designs, such as non-randomized approaches. Based on the analysis of existing clinical studies discussed in the preceding section and considering the established design criteria for clinical studies, it is crucial that clinical studies investigating the correlation between ART and the risk of breast cancer adhere to the following criteria. In the experimental design, the inclusion of a control group is of utmost importance. The control group should not only consist of individuals from the general population but also encompass infertile patients who have not undergone any form of fertility treatment. This criterion can be attributed to the absence of protective factors typically associated with pregnancy in infertile patients, resulting in a significantly higher susceptibility to breast cancer when compared to women who do not experience infertility^156^. Furthermore, in addition to considering the number of participants and the duration of follow-up, researchers must prioritize the inclusion of comprehensive clinical data. This includes accounting for confounding factors and factors associated with ART treatment to maintain the validity and reliability of the study^25^. Once the study has commenced, the researcher must exercise discretion in ensuring that the study group and the control group are subjected to the same level of follow-up. Additionally, the researcher must be mindful of the duration of the study's follow-up period and make efforts to minimize subject dropout. In the field of data analysis, it is of utmost importance to meticulously deliberate the choice of appropriate risk measures. This entails addressing errors factors to mitigate the potential influence of extraneous variables on the outcomes of the study^125^, ^169^. It is also advantageous to perform comprehensive subgroup analyses to monitor susceptible subgroups, thereby contributing to the examination of risk heterogeneity and precision medicine^162^.

In addition, it is crucial to perform thorough examinations on the breast tissue of patients who have undergone ART. These examinations should encompass a comprehensive range of analytical levels, comprising molecular, microenvironmental, and tissue assessments. Besides, it is imperative to investigate comparable therapeutic approaches on breast cells or animal models that replicate the hormonal and structural changes encountered by individuals after undergoing ART treatment. At the genetic and molecular biology level, researchers possess the capacity to perform transcriptional analysis on breast tissue samples acquired from patients. By comparing these samples with those obtained from infertile women and women with normal pregnancies, researchers can examine potential changes in chromosomal and genetic damage, as well as signaling pathways, after treatment^68^, ^101^, ^170^, ^171^. At the microenvironmental level, it is imperative to detect reproductive hormones and their metabolites in the breast tissue of patients undergoing ART treatment. This is particularly important in identifying toxic substances that are closely associated with cancer development, as well as cytokines like HIF-1^62^, ^77^, ^108^, ^126^. At the tissue level, it is advantageous to identify epigenetic remodeling of mammary cells and alterations in the structure of mammary tissue to ascertain the predisposition to breast cancer^77^, ^78^, ^93^, ^172^. While the alterations in the body resulting from ART are highly intricate and unpredictable, there remains a demand for replication of the modifications in the body's internal milieu induced by ART as closely as feasible in *in vitro* cellular and animal experiments^173^, ^174^. To enhance the comprehensiveness of the knowledge regarding the association between ART and the potential risk of breast cancer, future investigations must incorporate more comprehensive patient testing and fundamental experiments.

**Figure 4:**
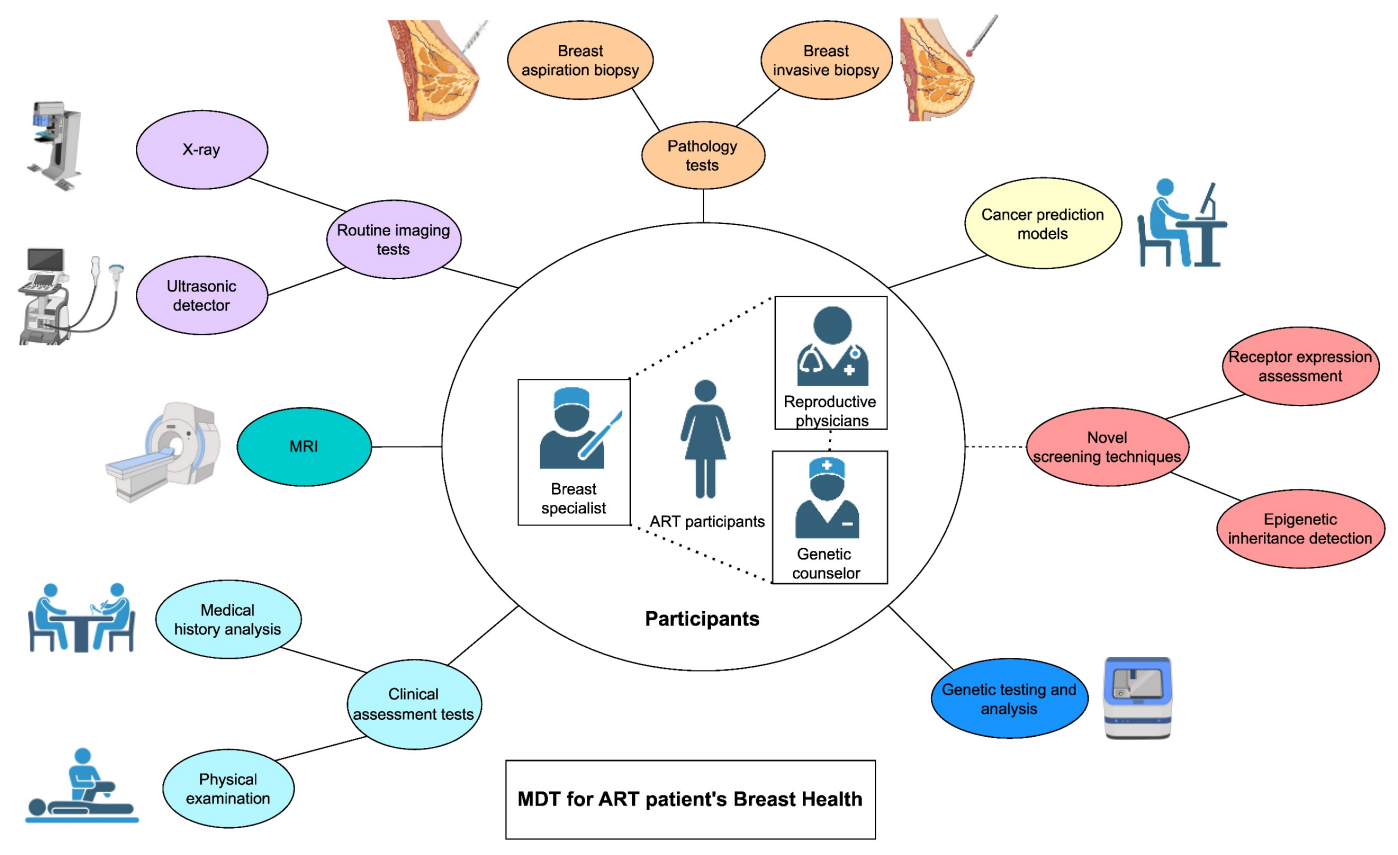
Breast health care for ART patients. An MDT typically involves a gathering of doctors or clinician scientists from various disciplines to deliberate on the clinical issues of a specific patient. The aim is to improve the resolution of complex clinical problems and offer more personalized and comprehensive medical guidance to the patient. This diagram illustrates the potential content and procedures of an MDT for the patient in question. For ART patients who face significant additional risk factors for breast cancer, an MDT involving a reproductive physician, breast specialist, and genetic counselor is beneficial. This process would include conventional tools such as clinical evaluation, imaging, and pathology, as well as novel tools like cancer prediction modeling and genetic testing. Some innovative tests that are not yet in clinical use are also included, and they may yield unexpected results for patients undergoing ART. MDT, multidisciplinary team; ART, assisted reproductive technology; MRI, magnetic resonance imaging.

According to the latest research findings, there is currently no conclusive evidence establishing a direct correlation between ART and the incidence of breast cancer. Therefore, it is not recommended for clinicians to make any specific modifications to breast health care or treatment plans for patients who have undergone ART. Of course, when a patient who is either on the verge of undergoing or has already undergone ART expresses apprehensions regarding the potential risk of developing breast cancer in the future, it is imperative that the physician offers a comprehensive explanation to the patient^175^. This explanation should highlight the absence of a conclusive correlation between ART and breast cancer and temper the psychological burden experienced by patients. If, based on future extensive clinical and basic science research, it is established that ART actually does increase the risk of breast cancer in patients, this finding should not be a cause for increased concern either. Breast cancer is commonly acknowledged as a multi-strike ailment that encompasses various factors and mechanisms in its pathogenesis, and it is imperative to acknowledge that no individual risk factor in isolation presents a substantial peril to the well-being of patients^176^. Simultaneously, there are established protocols and clinical programs that have been specifically developed for women who have a higher risk of developing breast cancer in comparison to the general population. In instances where patients exhibit high-risk factors, it is advisable to adopt a more rigorous approach to screening for breast disease, including reducing the time intervals between screenings and contemplating the utilization of additional diagnostic tests^177^-^180^. Relevant investigations encompass a range of clinical assessment methods, laboratory tests, imaging tests, pathology tests, and other related procedures^181^, ^182^. Especially for ART patients with significant risk factors for breast cancer, such as advanced age, a family history of cancer, or the presence of precancerous lesions, it is advisable to facilitate a multidisciplinary team (MDT) discussion and consultation^183^. This collaborative effort should involve reproductive physicians, breast specialists, and genetic counselors to ensure the preservation of patients' breast health and effectively manage any potential risks related to breast cancer. Additionally, breast professionals can recommend the utilization of advanced techniques such as X-ray and ultrasound^184^. Furthermore, breast specialists and oncologists may consider incorporating the BOADICEA model in the assessment of patients who have undergone ART treatment and exhibit multiple risk factors^185^. When conditions permit, it is also essential and advantageous to undergo testing for cancer susceptibility genes and to receive risk counseling. For patients with suspected precancerous lesions in the breast that have already been discovered, it is possible to undergo a pathological test of the breast lesions before receiving ART treatment^186^.(Fig. 4) Researchers who are interested in this field can further explore these methods through comprehensive investigations, which may ultimately provide benefits to all breast cancer patients and individuals at a heightened risk of developing breast cancer.

## 6. Conclusions and future perspectives

Societal progress has coincided with a growing prevalence of infertility among women. Assisted reproductive technology (ART) has emerged as an essential therapeutic approach for individuals seeking to realize their aspirations of parenthood. Nevertheless, the increasing concern among patients and clinicians regarding the potential correlation between and the risk of breast cancer is primarily driven by the perceived association between reproductive hormones and this disease. From a theoretical standpoint, the administration of ART treatment inherently entails the exposure of patients to elevated concentrations of reproductive hormones, including estrogen and progesterone, along with their corresponding metabolites, within a condensed timeframe. This alteration appears to be correlated with an elevated susceptibility to the development of breast cancer, which has been demonstrated in previous literatures. However, the current body of clinical evidence does not provide robust support for this assertion. Our comprehensive analysis of recent clinical studies reveals that although certain cohort studies demonstrate a potential positive correlation between ART treatment and breast cancer, further investigation indicates that the association remains uncertain, and there is even evidence suggesting that ART treatment might mitigate the risk of developing breast cancer. Therefore, based on the analysis of clinical studies, it can be affirmed that there is no conclusive evidence supporting a direct association between ART treatment and the incidence of breast cancer at this time.

Despite the perplexing nature of this conflict, the association between ART and the risk of breast cancer remains ambiguous. However, we have also observed relatively positive indications, indicating that there is no definitive correlation between ART and the incidence of breast cancer in patients undergoing treatment. Even if a potential correlation between the mentioned risk factors is substantiated in the future, it is important to note that the level of risk is relatively low; thus, it is probably unlikely to represent a significant burden to the overall breast health of patients. This might reduce any unwarranted anxiety about potential negative effects of treatment on breast health between the patient and the reproductive specialist before the patient undergoes ART. In the meantime, more comprehensive and standardized clinical studies are required to establish higher-level evidence, and it is also imperative that advances in corresponding basic research be made. It is anticipated that forthcoming research will provide further insight into the association between and the risk of developing breast cancer. Additionally, it is expected that this research will shed light on the alterations occurring in breast tissue and the potential underlying mechanisms involved in this process. Conclusive findings hold the potential to mitigate the anxieties and errors in clinical decision-making that arise from ambiguous information, benefiting both patients and healthcare professionals in the context of ART.

## Supplementary Material

Supplementary figures and table.

Supplementary search strategy.

## Figures and Tables

**Table 1:** Factors Associated with Breast Cancer Development

Categories	Categories			Incidence
Risk factors	Non-hormone related		Hereditary	The risk of breast cancer is 250% higher for individuals diagnosed in the immediate family and 50% higher for those diagnosed in the non-immediate family.^4^
			Germline mutations	Women carrying the BRCA1/2 gene exhibit a cumulative risk of breast cancer development that surpasses twice the risk observed in the general population.^11^
			Smoking	Women who are current smokers have a 24% higher risk of breast cancer than non-smokers, and women with a history of previous smoking have a 13% higher risk of breast cancer than non-smokers.^6^
			Chest radiotherapy	Patients who received a radiation dose of 4 Gy or more to the breast had a nearly threefold increased risk of developing breast cancer compared to those who did not undergo radiation therapy.^7^
			Alcohol	Women who consume alcohol 2-3 times a day have a 20% higher risk of developing breast cancer compared to those who abstain from alcohol.^10^
	Hormone related	Endogenous	Menstruation period	Earlier age at menarche (less than 12 years) increases the risk of breast cancer by a factor of 1.050, while later age at menopause (greater than 55 years) increases the risk of breast cancer by a factor of 1.029.^15^
		Exogenous	OC	Recent use of oral contraceptives (within the past year) was associated with a 50% higher risk of breast cancer compared to never using them or using them in the past.^18^, ^19^
			HRT	Receiving hormone replacement therapy increases the risk of breast cancer by more than 40 percent. Additionally, women who use a combination of estrogen and progestin therapy have a higher incidence of breast cancer compared to those who use estrogen-only therapy.^18^, ^19^
Protective factors			Nutritional supplementation	Recent use of oral contraceptives (within the past year) was associated with a 50% higher risk of breast cancer compared to never using them or using them in the past.^5^
			First childbirth	Women in the youngest age at first birth category had a 27% lower risk of HR+ breast cancer than women in the highest age at first birth category.^16^
			Breastfeeding	Women in the youngest age at first birth category had a 27% lower risk of HR+ breast cancer than women in the highest age at first birth category.^16^
			Exercise	Women who engage in regular exercise have a 10-20% lower risk of developing breast cancer.^9^
			Obesity	Premenopausal obese women have a more than 20 percent lower risk of breast cancer, while postmenopausal obese women have a 70 percent higher risk of breast cancer.^8^

Gy, Gray; HR, hormone receptor; BRCA, breast cancer susceptibility gene

**Table 2A:** Clinical researches on ART

Authors	Publication years	Designs	Districts	Periods of study	Populations	Following up years	Adjusting factors	Type of infertility treatment	Cycles
Lundberg *et al.*^140^	2016	Cross-sectional	Sweden	2010-2013	Total: 43,313		AgeFertility statusSmoking and DrinkingFamily history of breast cancer	Hormonal stimulationCOS	
Machtinger *et al.*^148^	2022	Cohort	Israel	1994-2019	Case: 32,366Control: 32,366	9.1 years -mean time	AgeFertility statusSmoking	UrinaryRecombinant	1-≥8
Perri *et al.*^147^	2021	Cohort	Israel	1995-2019	Total: 1,824 Case: 332 Control: 1,492	86,065 person years-total	Age Fertility status OCP and HRT	Clomiphene citrate Gonadotropins IVF	
Vassard *et al.*^33^	2021	Cohort	Denmark	1994-2016	Case: 61,579Control: 579,760	Case: 9.69 years Control: 9.28 years	AgeFertility statusFamily history of breast cancer	Programs of access of ART	1-≥5
Tsafrir *et al.*^129^	2020	Cohort	Israel	1994-2002	Total: 501	16.7 years -mean time	AgeFertility status	IVF	1-≥4
Derks-Smeets *et al.*^146^	2018	Cohort	Netherlands	2010-2013	Total: 2,514		Age	IVF	
Williams *et al.*^139^	2018	Cohort	UK	1991-2002	Total:255,786	2,257,789 person years-total8.8 years-mean time	AgeFertility statusHistory of cancer disease		1.8 in average
Reigstad *et al.^138^*	2017	Cohort	Norway	1960-1996	Total: 1,353,724 Case: 56,194 Control: 1,297,530	12,354,392 person years-total 11.0 years -mean time	Age Fertility status	IVF Clomiphene citrate Other medications	1-≥6
Lundberg *et al.*^141^	2017	Cohort	Sweden	1982- 2012	Total:1,340,211	Case: 9.6 years Control: 14.6 years	Age ParityAge at first birth Family history of breast cancer	Clomiphene citrateGonadotropins	
Kessous *et al.*^151^	2016	Cohort	Israel	1988- 2013	Total:10,6031Case:4,363Control:101,668	11.6 years -mean time	AgeFertility statusObesity	OIIVF	
van den Belt-Dusebout *et al.*^150^	2016	Cohort	Netherlands	1980-1995	Total: 25,108Case: 19,158	21.1 years -median time	AgeFertility statusFamily history of breast cancer	IVF	1-≥7
Reigstad *et al.*^130^	2015	Cohort	Norway	1984- 2010	Total:808,834Case: 16,626Control: 792,208	12,401,121 person years-total16.0 years -mean time	Age Fertility status Region of residence	IVF、ICSI	
Luke *et al.*^142^	2015	Cohort	USA	2004-2009	Total:113,226Case: 59,354Control: 53,872	263,457 person years-total4.8 years-mean time	Age Fertility statusCumulative FSH dosage	FSH Clomiphene citrate	1, 2, 3, 4, or ≥5
Brinton *et al.*^123^	2014	Cohort	USA	1965-1988	Total: 9,892	285,332 person years-total30.0 years -median time	AgeFertility statusFamily history of breast cancer	ClomipheneGonadotrophins	<6->12
Brinton *et al.*^143^	2013	Cohort	Israel	1994-2011	Total: 87,403	704,241 person years-total8.1 years -mean time	AgeFertility statusSmoking and Drinking	IVF including (GnRH analogues, Clomiphene, Progestogen)	1-≥4
Lerner-Geva *et al.*^131^	2012	Cohort	Israel	1964- 1974	Total:2,431	88,186 person years-total33.8 years -mean time	Age	Clomiphene citratehMG	
Stewart *et al.*^44^	2012	Cohort	Australia	1983-2002	Total: 21,025Case:7,381Control: 13,644	16.3 years -mean time	AgeFertility statusRace	IVF	
Yli-Kuha *et al.*^152^	2012	Cohort	Finland	1996-2004	Total: 18,350Case: 9,175Control: 9,175	7.8 years -mean time	Socio-economic positionMarital status	IVF	
Källén *et al.*^145^	2011	Cohort	Sweden	1982- 2006	Case:24,058Control: 1,394,061		Age Fertility status Smoking	IVF	
Silva Idos *et al.*^132^	2009	Cohort	UK	1963- 1999	Total: 9,152Case:7,425Control: 1,727	21.4 years -mean time	Age Fertility status	Clomiphene citrate Gonadotropins	2-3
Calderon-Margalit *et al.*^133^	2009	Cohort	Israel	1974- 2004	Total:14,463	424,193 person years-total29.0 years -mean time	AgeSocioeconomic status Geographic origin Fertility status	Clomiphene citrate	
Orgéas *et al.*^153^	2009	Cohort	Sweden	1961- 2004	Total:1,135	35,092 person years-total30.9 years -mean time	Age Fertility status	Clomiphene citrate Gonadotropins	1-≥4
Pappo *et al.*^125^	2008	Cohort	Israel	1986- 2003	Total:3,375	27,327 person years-total8.1 years -mean time	Age Fertility statusFamily history	IVF	1-18
Kristiansson *et al.*^134^	2007	Cohort	Denmark	1965-1998	Total:54,379	8.8 years -mean time	Fertility status	FSHhCG hMGGnRH	
Jensen *et al.*^154^	2007	Cohort	Sweden	1981- 2001	Case: 8,716Control: 64,0059	Case: 6.2 years Control: 7.8 years	AgeFertility status	IVF of COH	
Lerner-Geva *et al.*^135^	2006	Cohort	Israel	1964-1984	Total: 120,895Case: 5,788	13.1 years -mean time	Age Fertility statusUse of oral contraceptives	Clomiphene citratehMG	1-≥6
Gauthier *et al.*^128^	2004	Cohort	France	1990- 2000	Total:92,555 Case: 6,602 Control:85,953	9.7 years -mean time	Smoking BMI Self and familial history of breast cancer Age at menarche Menopausal status Fertility status	Clomiphene citrate Gonadotropins	
Brinton *et al.*^136^	2004	Cohort	USA	1965-1988	Total: 12,193	18.8 years -mean time	Age Fertility statusFamily history of breast cancer	ClomipheneGonadotrophins	<6->12
Lerner-Geva *et al.^155^*	2003	Cohort	Israel	1984-1996	Total: 1,082Case: 5	6.5 years -mean time	Fertility status	IVF	1-≥6
Taheripanah *et al.^149^*	2018	Case-control	Iran	2011-2013	Case: 928Control: 928		AgeFertility statusFamily history of breast cancer OCP and HRT	Clomiphene citratehCG	less or more than 6 months
Fei *et al.*^144^	2012	Case-control	Worldwide	2008-2010	Total:3,091Case:1,422Control: 1,669		AgeFertility statusSmoking and Drinking	Clomiphene citrateFSH	
Burkman *et al.*^137^	2003	Case-control	USA	1994- 1998	Case: 4,575 Control: 4,682		Age RaceStrata of study center	Clomiphene, hMG	<6-≥6

**Table 2B:** Clinical researches on ART, detailed

Estimating data	Conclusions	Possible errors
Women with a history of infertility had 1.53 cm^3 higher absolute dense volume compared to non-infertile women (95% CI 0.70-2.35). Among infertile women, only those who had gone through COS treatment had a higher absolute dense volume than those who had not received any hormone treatment (adjusted MD=3.22, 95% CI 1.10-5.33).	There was higher absolute dense volume in women treated with COS, which may indicate higher cancer risk in infertile women who undergo COS.	C (The intentions for fertility medicines, the number of cycles of ART treatment, and the infertility diagnosis were not made clear.)G (There may be a chance of misclassification because the study relied on self-reports of infertility and fertility treatments.)
The incidence rates of breast cancer per 10,000 person years were 11.9 (95% CI, 10.7-13.3) and 10.7 (95% CI, 9.6-12.0) in the ART group and general population, respectively. The adjusted risk for breast cancer was similar in the ART group compared with the general population (HR=1.10, 95% CI 0.94-1.28). And among women diagnosed with breast cancer, the prevalence of BRCA1/2 mutations and tumor staging did not differ between the ART and general population groups.	The risk of breast cancer among women treated by ART was similar to the risk among women who did not undergo fertility treatments.	C (There was no way to subclassify exposed patients in the database based on the cause of their infertility)D (Not having certain breast cancer risk factors, such as a family history, taking oral contraceptives, getting hormone replacement treatment, etc.)H (The average age of patients with breast cancer was 42 years old, therefore the individuals were still reasonably young. Since 10% of breast cancer occurrences occur in those over 42, the study is unable to determine if ART influences the risk of breast cancer in the remaining 90% of cases.)
The study findings indicated that there was no significant correlation between the risk of breast cancer and the administration of assisted reproductive technology (ART) treatment (HR=1.00, 95% CI 0.81-1.22). The study findings indicate that there was an increased risk of breast cancer in women who were exposed to clomiphene citrate (HR=1.12; 95% CI 0.93-1.35), particularly in women who had given birth (HR=1.26; 95% CI 1.03-1.54).	The administration of ART is not generally linked to an elevated risk of breast cancer, while the administration of clomiphene citrate has been found to increase the risk of breast cancer.	C (The study was susceptible to potential misclassification of exposure due to the limited scope of the database, which only encompasses data from 2004.) D (Insufficient family history information and the use of other treatments like OC) H (The participants in the study were relatively young, even at the conclusion of the study period.)
5861 women were diagnosed with breast cancer, 695 among ART-treated and 5166 among untreated women (1.1% versus 0.9%, P < 0.0001), while after using Cox regression adjusted analyses, the risk of breast cancer was slightly increased among women treated with ART (HR=1.14, 95% CI 1.12-1.16).The risk of breast cancer increased with higher age at ART treatment initiation and was highest among women initiating treatment at age 40þ years (HR=1.37, 95% CI 1.29-1.45).	There is a slightly increased risk of breast cancer in women who underwent ART treatment, and also an increased risk of breast cancer associated with a higher age at ART treatment initiation has been shown.	C (This study was unable to separate the possible impact of ART treatment on breast cancer risk from infertility since it did not include a reference group of infertile women not receiving ART treatment.)G (The study's limitations were the inability to discriminate between different subtypes of breast cancer and the exclusion of ductal carcinoma in situ as an outcome owing to national policy.)
22 women were diagnosed with invasive breast cancer, compared with 19.84 expected (SIR=1.11, 95% CI 0.69-1.68).	Older women (≥40) undergoing IVF treatment were not significantly associated with an excess risk of cancer at long-term follow up.	A (The overall sample was definitely small, as accessed from only two medical centers rather than being population based)C (No available treatment programs of IVF)D (There was a lack of all the breast cancer risk factors when considering adjusting factors as the dataset's deficiency)H (Use the SIR as a statistical parameter, and fail to consider other factors that influence breast cancer risk.)
Of the 2514 BRCA1/2 mutation carriers, 76 were exposed to ovarian stimulation for IVF, and 938 BRCA1/2 mutation carriers were diagnosed with breast cancer. IVF exposure was associated with risk of breast cancer (HR=0.79, 95%CI 0.46-1.36). Similar results were found for the subgroups of subfertility women (n=232; HR=0.73, 95% CI 0.39-1.37).	It was found for a negative association between ovarian stimulation for IVF and breast cancer risk in BRCA1/2 mutation carriers.	A (Since fewer women were subjected to IVF overall, the study's power was nevertheless constrained by the small sample size.)C (Since data were self-reported, exact details on the regimens and cycles utilized in IVF were absent)D (When taking into account modifying variables, there was a deficiency in all breast cancer risk factors, partially owing to recollection bias resulting from the investigation's methods).G (Survival bias may have arisen from the retrospective research design if women exposed to IVF had tumors with a poorer prognosis)
There is no significant change in risks of breast cancer overall (SIR=0.98, 95% CI 0.94-1.01) or invasive breast cancer SIR=0.96, 95% CI 0.92-1.00), while an increased risk of in situ breast cancer (SIR=1.15, 95% CI 1.02-1.29)	No association between ART and invasive breast cancer risk, but increased risks of in situ breast cancer	C (No ART treatment programs available)H (Use the SIR as a statistical parameter and neglect to take into account additional variables that can directly affect the risk of breast cancer from the entire set of data)
The study findings indicated that there was no significant correlation between the risk of breast cancer and the administration of assisted reproductive technology (ART) treatment (HR=1.00, 95% CI 0.81-1.22). The study findings indicate that there was an increased risk of breast cancer in women who were exposed to clomiphene citrate (HR=1.12; 95% CI 0.93-1.35), particularly in women who had given birth (HR=1.26; 95% CI 1.03-1.54).	The administration of ART is not generally linked to an elevated risk of breast cancer, while the administration of clomiphene citrate has been found to increase the risk of breast cancer.	C (The study was susceptible to potential misclassification of exposure due to the limited scope of the database, which only encompasses data from 2004.) D (Insufficient family history information and the use of other treatments like OC) H (The participants in the study were relatively young, even at the conclusion of the study period.)
The risk of breast cancer in women who gave birth after ART compared with women who gave birth after spontaneous conception were exhibited as follow (adjusted HR=0.84; 95%CI 0.74-0.95).	Women treated with ART had a lower risk for breast cancer	C (Fail to ascertain the number of ART cycles each woman had gone through)
A total of 528 patients developed breast cancer during the follow-up period. The incidence of breast cancer was 0.4% among those treated with IVF (n=1149), 0.5% among those treated with OI (n=3214), and 0.4% among those not treated with ART. A t-test was conducted, yielding a p-value of 0.926, indicating no significant differences were found.	No significant association was found between fertility treatments (OI and IVF) and future risk of breast cancer.	D (Lack of knowledge about parity, family history, and awareness on other treatments like OC)H (The effect of other effects on patients who underwent ART compared to other patients could not be excluded because this study only used t-tests for patients who acquired breast cancer.)
Breast cancer risk in IVF-treated women was not significantly different from that in the general population (SIR=1.01, 95% CI 0.93-1.09) and from the risk in the non-IVF group (HR=1.01, 95% CI 0.86-1.19). The SIR did not increase with longer time since treatment (≥20 years) in the IVF group (SIR=0.92, 95% CI 0.73-1.15) or in the non-IVF group (SIR=1.03, 95% CI 0.82-1.29]).	The finding is consistent with absence of a significant increase in long-term risk of breast cancer among IVF-treated women.	D (A number of possible confounding variables had high missing data rates, and there was an imbalance with the non-IVF group having 33% more missing data than the IVF group (16%))G (Because cancer incidence was only known for responding women before to 1989 and not for nonresponding women due to statistical limitations, some instances may have gone unnoticed.)
Compared with controls, an HR of 1.20 (95% CI 1.01-1.42) for women treated with IVF 1.35 (95% CI 1.07-1.71) for women with follow-up >10 years	Increased risk of breast cancer in women with ART.	C (The number of cycles of ART treatment and the diagnosis of infertility were not specified.)
Women treated with ART had a lower risk for breast cancer (for all women: SIR=0.83, 95% CI 0.75-0.91; women without prior ART: SIR=0.77, 95% CI 0.66-0.89)	Women treated with ART had a lower risk for breast cancer	E (Only 4.8 years were followed up on)H (Use the SIR as a statistical metric; no specific risk factor information is available, but the research analyzes the HR of several adjusting factors.)
Ever use of clomiphene citrate was not associated with risk (HR=1.05, 95% CI 0.90-1.22) vs. never use. While ever use of gonadotrophins was slightly associated with risk (HR=1.14, 95% CI 0,89-1,44) vs. never use, and a significant relationship of use gonadotrophins with invasive cancers was seen among women who remained nulligravid (HR=1.98, 95% CI, 1.04-3.60).	Fertility drugs stimulating ovulation are not associated with increased risk for breast cancer, but use of gonadotrophins was slightly associated.	D (Not all women's possible confounders were included in the investigation.)F (There are comparatively few occurrences of breast cancer)H (Some of the derived risks had poor precision, especially within subgroups, and the subjects were still relatively young (52.7 years on average for patients with breast cancer).)
There was a slightly decreased alteration in breast cancer risk among women who had received fertility treatment compared with those who received no treatment (HR=0.87, 95% CI 0.71-1.06).	There were a slightly negative relationships of IVF exposures to the risks of breast cancers.	C (Only roughly half of the study participants had information on the etiology of infertility.)D (Some information about other known risk factors, such as a family history of cancer and the use of oral contraceptives, was lacking.)
Women treated for infertility had a higher risk for breast cancer (SIR=1.1, 95% CI 0.98-1.36)	Women treated for infertility had a borderline increased risk for breast cancer.	A (The sample size was tiny overall.)C (The precise number of ART treatment cycles was not disclosed.)D (Inadequate knowledge about family history, parity, and other treatments like OC)F (There are comparatively few occurrences of breast cancer)H (Use the SIR as a statistical measure without considering additional variables that affect the risk of breast cancer.)
There was no overall increase in the rate of breast cancer in women who had IVF (HR=1.10, 95% CI 0.88-1.36)There was an increased rate in women who commenced IVF at a young age (younger than 24 years old) (HR=1.59, 95% CI 1.05-2.42)	There was no overall increase in the rate of breast cancer in women who had IVF, while commencing IVF treatment at a young age was associated with an increased rate of breast cancer.	A (The sample size was tiny overall.)C (It was not possible to determine the number of IVF treatment cycles or the kinds or dosages of fertility medications.)D (Not having certain risk factors for breast cancer, such as a family history, using oral contraceptives, getting hormone replacement therapy, etc.)
There were 115 breast cancer cases in general population and 55 breast cancer cases in exposed (OR=0.93, 95% CI 0.62-1.40)	The breast cancer incidence was similar among IVF women and controls.	A (The sample size was tiny overall.)C (There was no sign of infertility, and the dosage and number of treatment cycles with the prescribed medicine were unclear.)D (When taking into account modifying factors, not all breast cancer risk factors were present.)E (Short follow-up)F (There are comparatively few occurrences of breast cancer)
Women used IVF had a lower risk for breast cancer compared with other women who have an infant during the observation period (OR= 0.76, 95% CI 0.62-0.94)	Decreased risk of breast cancer in women with IVF treatment.	C (The precise number of ART treatment cycles was not disclosed.)D (Lack of knowledge of alternate treatments, such as OC, and family history)E (Accurate follow-up time information is not provided.)
Women treated with ovarian stimulation drugs had a higher risk for breast cancer (RR=1.15, 95% CI 0.80 - 1.68)Relative to the general population, the cohort experienced higher incidence of breast cancer (SIR=1.13, 95% CI 0.97-1.30)	Data shows that women treated with ovarian stimulation drugs had a higher risk for breast cancer.	C (Do not know the diagnosis of infertility)D (No information pertaining to family history)F (There are comparatively few occurrences of breast cancer)
Women treated with ovulation induction had a higher risk for breast cancer (multivariate HR=1.42, 95% CI 0.99, 2.05)	Ovulation induction was associated with a borderline-significant increased risk of breast cancer, and women who used drugs to induce ovulation had increased risks of cancer at any site.	C (Details about the kind of infertility, the kind of treatment, and the number of cycles were lacking.)D (Lack of knowledge of alternate treatments, such as OC, and family history)
The study showed no overall alternation in the risks for breast cancer with women under any exposure to hormonal fertility treatment (rates adjusted, SIR=1.01, 95% CI 0.77-1.31)	No overall association between fertility drugs and breast cancer risk.	A (The sample size was tiny overall.)F (There are comparatively few cases of breast cancer, even fewer than 100)H (Use the SIR as a statistical metric without taking into account various other variables that affect the risk of breast cancer.)
Among 3,375 IVF-treated women, 35 breast carcinomas were diagnosed compared to 24.8 cases expected (SIR=1.4, 95% CI 0.98-1.96), which shew increased risk of breast cancer in women who used fertility treatmentMultivariate analysis revealed that women who underwent >or=4 IVF cycles compared to those with one to three cycles were at risk to develop breast cancer, although not significantly (SIR = 1.9, 95% CI 0.95-3.81).	There is probably an increased risk of breast cancer in women who used IVF treatment, and women who underwent more IVF cycles may experience higher risk.	A (There was undoubtedly a limited sample size overall)E (The follow-up period is comparatively insufficient)F (There are comparatively few cases of breast cancer—less than 100 cases—in the world.)
The study showed no overall increased breast cancer risk after use of fertility drugs (RR=0.94-1.28 according to different drugs), whereas use of progesterone increased breast cancer risk (RR=3.36, 95%CI 1.3-8.6)	: The results showed no strong association between breast cancer risk and use of fertility drugs, while use of progesterone increased breast cancer risk significantly.	C (The number of cycles of ART treatment and the diagnosis of infertility were not specified.)D (All non-research factor information is missing, with the exception of parity)F (There are comparatively few occurrences of breast cancer)
In a multivariate Poisson regression analysis, adjusted RR of 0.93 (95% CI 0.58-1.43) among IVF women was found for the risk of carcinoma in situ (CIS) of the breast cancer	The women who underwent IVF treatment had no alternation of breast cancer.	C (The precise number of ART treatment cycles was not disclosed.)D (No information was provided for smoking, the use of oral contraceptives, or the incidence of cancer in families.)E (The follow-up period is comparatively insufficient)
Compared to 115.2 expected breast cancer cases, 131 cases were observed (SIR=1.1, 95% CI 0.9-1.4). Risk for breast cancer was significantly higher for women treated with clomiphene citrate (SIR=1.4, 95% CI 1.0-1.8).	Infertility and usage of infertility drugs are not associated with increased risk for breast cancer in general. However, breast cancer risk is elevated when treated with clomiphene citrate.	D (Lack family history information)G (The age at which breast cancer incidence peaks is not yet the mean age at the end of the follow-up).H (Use the SIR as a statistical metric without considering various other variables that affect the risk of breast cancer.)
The study showed no overall association between breast cancer risk and treatment of ART (RR=0.95, 95% CI 0.82-1.11)	No association between ART treatment and breast cancer.	C (The number of cycles of ART treatment and the diagnosis of infertility were not specified.)
Infertile patients had a significantly higher breast cancer risk than the general population (SIR=1.29, 95% CI 1.1-1.4). The cohort showed clomiphene adjusted RR is 1.02(95% CI 0.8-1.3) and gonadotrophins adjusted RR is 1.07(95% CI 0.7-1.6).When seen after > or = 20 years of follow-up, the cohort showed clomiphene adjusted RR=1.39 (95% CI 0.9-2.1) and gonadotrophins adjusted RR=1.54 (95% CI 0.8-3.2).	There was no overall increase in breast cancer risk associated with use of ovulation-stimulating drugs, while slight and non-significant elevations in risk were seen for both drugs after > or = 20 years of follow-up.	D (A handful of women's workups were incomplete, which left the causes of their infertility unclear)G (Twenty percent of the study participants could not be located, and an additional eleven percent did not grant permission to view their medical data).
5 cases of breast cancer were observed as compared to 4.88 that were expected (SIR=1.02, 95% CI 0.33-2.39)	Infertility treatment is not associated with increased risk for breast cancer.	A (There was undoubtedly a limited sample size overall)C (The number of ART treatment cycles and the planned for fertility medicines were not made clear.)D (When taking into account modifying factors, the majority of the breast cancer risk factors were absent.)E(The follow-up was rather brief)F (Even five cases of breast cancer were reported, indicating a certain low number of cases)H (Use the SIR as a statistical metric without taking into account additional risk variables that affect the chance of developing breast cancer)
The use of ovulation induction drugs was not significantly associated with an increased risk of breast cancer (OR=1.13, 95% CI 0.7-1.85) among women with infertility (OR=1.28, 95% Cl 0.8-1.95).	There was no statistically significant relationship between infertility and ovulation induction drugs with the risk of breast cancer	B (The sample size was rather modest overall.)C (The fact that the modifications had no effect on the total estimates suggests that these risk factors are not confounding the relationship between the use of reproductive medications and breast cancer risk.)
Women who had used fertility drugs showed a non-statistically significantly decreased risk of breast cancer overall compared with nonusers (OR=0.82, 95% CI 0.63-1.08).	The risk of breast cancer among women treated by ART was decreasing compared with nonusers.	B (The sample size was rather modest overall.)C (The information about infertility diagnosis is lacking and the fertility-drug use is self-reported.)D (On average, the case sisters were younger than the control sisters.)G (At least a year passes following diagnosis; hence, some case sisters with more serious cancers passed away before contacting.)
For all the women, infertility drugs were not associated with an overall increased risk of breast cancer (OR=0.9, 95% CI 0.8-1.2). However, the relative risk of breast cancer was substantially higher for women who used hMG for at least 6 cycles, ranging from 2.7 to 3.8.	A history of overall infertility drug use was not associated with the risk of developing breast cancer, while long-term use of hMG could adversely affect risk of breast cancer.	B (There were only 28 cases in the sample of patients with hMG, which was much smaller than the whole sample size).C (Do not know the diagnosis of infertility)D (Insufficient knowledge about reproductive status)

**Table 3:** Related experiments

Levels		Experimental Types
		
Cellular level		Signaling pathways
		Cellular Epigenetic Assays
		Single-cell sequencing
		Cellular Epigenetic Assays
		
Tissue level		Microenvironmental Analysis
		Histopathology
		Tissue Transcription Analysis
		Animal and Organoid Models
		
Clinical Level		Prospective Studies
		Retrospective Studies
		Subgroup Analysis
		Meta-analysis

## Abbreviations

ART: Assisted Reproductive Technology
BRCA: Breast Cancer Susceptibility Gene
cAMP: Cyclic Adenosine Monophosphate
COH: Controlled Ovarian Hyperstimulation
COMT: Catechol-O-Methyltransferase
CYP: Cytochrome P450 Proteins
DR4: Death Receptor 4
E1: Estrone
E2: Estradiol
ECM: Extracellular Matrix
EMT: Epithelial-Mesenchymal Transition
ER: Estrogen Receptor
ET: Embryo Transfer
FACS: Fluorescence-Assisted Cell Sorting
FSH: Follicle-Stimulating Hormone
GnRH: Gonadotropin-Releasing Hormone
GSTP1: Glutathione S-Transferase P1
HCG: Human Chorionic Gonadotropin
HIF-1: Hypoxia-Inducible Factor-1
HMG: Human Menopause Gonadotropin
HRT: Hormone Replacement Therapy
HR: Hazard Ratio
ICSI: Intracytoplasmic Single-Sperm Injection
IF: Impact Factor
IVF: *In Vitro* Fertilization
MAPK: Mitogen-activated Protein Kinase
MD: Mean Deviation
MDT: Multidisciplinary Team
MeSH: Medical Subject Headings
MRI: Magnetic Resonance Imaging
NF-ĸB: Nuclear Factor-Kappa B
NRF1: Nuclear Respiratory Factor-1
OC: Oral Contraceptive
OHSS: Ovarian Hyperstimulation Syndrome
OR: Odds Ratio
PGT: Preimplantation Genetic Testing
PI3K: Phosphatidylinositol 3-Kinase
PR: Progesterone-Receptor
PRL: Prolactin
Q: Quinone
RCT: Randomized Controlled Trial
ROS: Reactive Oxygen Species
RR: Relative Risk
SIR: Standardized Incidence Ratio
Sp1: Specificity Protein 1
SQ: Semiquinone
SULT: Sulphotransferase
VEGF: Vascular Endothelial Growth Factor
WHO: World Health Organization
16α-OHE1: 16alpha-Hydroxy Estrone
2-OHE1: 2-Hydroxy Estrone
2-OHE1: 2-Hydroxy Estradiol
4-OHE1: 4-Hydroxy Estrone
4-OHE2: 4-Hydroxy Estradiol

## References

[1] Wilkinson L, Gathani T (2022). Understanding breast cancer as a global health concern. The British Journal of Radiology.

[2] Sung H, Ferlay J, Siegel RL, Laversanne M, Soerjomataram I, Jemal A (2021). Global Cancer Statistics 2020: GLOBOCAN Estimates of Incidence and Mortality Worldwide for 36 Cancers in 185 Countries. CA: a cancer journal for clinicians.

[3] Fitzmaurice C, Abate D, Abbasi N, Abbastabar H, Abd-Allah F, Abdel-Rahman O (2019). Global, Regional, and National Cancer Incidence, Mortality, Years of Life Lost, Years Lived With Disability, and Disability-Adjusted Life-Years for 29 Cancer Groups, 1990 to 2017: A Systematic Analysis for the Global Burden of Disease Study. JAMA oncology.

[4] Pharoah PD, Day NE, Duffy S, Easton DF, Ponder BA (1997). Family history and the risk of breast cancer: a systematic review and meta-analysis. International journal of cancer.

[5] Aune D, Chan DS, Vieira AR, Rosenblatt DA, Vieira R, Greenwood DC (2012). Fruits, vegetables and breast cancer risk: a systematic review and meta-analysis of prospective studies. Breast Cancer Res Treat.

[6] Gaudet MM, Gapstur SM, Sun J, Diver WR, Hannan LM, Thun MJ (2013). Active smoking and breast cancer risk: original cohort data and meta-analysis. Journal of the National Cancer Institute.

[7] Travis LB, Hill DA, Dores GM, Gospodarowicz M, van Leeuwen FE, Holowaty E (2003). Breast cancer following radiotherapy and chemotherapy among young women with Hodgkin disease. Jama.

[8] Rosner B, Eliassen AH, Toriola AT, Hankinson SE, Willett WC, Natarajan L (2015). Short-term weight gain and breast cancer risk by hormone receptor classification among pre- and postmenopausal women. Breast Cancer Res Treat.

[9] Friedenreich CM, Ryder-Burbidge C, McNeil J (2021). Physical activity, obesity and sedentary behavior in cancer etiology: epidemiologic evidence and biologic mechanisms. Molecular oncology.

[10] Suzuki R, Orsini N, Mignone L, Saji S, Wolk A (2008). Alcohol intake and risk of breast cancer defined by estrogen and progesterone receptor status-a meta-analysis of epidemiological studies. International journal of cancer.

[11] Kuchenbaecker KB, Hopper JL, Barnes DR, Phillips KA, Mooij TM, Roos-Blom MJ (2017). Risks of Breast, Ovarian, and Contralateral Breast Cancer for BRCA1 and BRCA2 Mutation Carriers. Jama.

[12] Guo Z, Zhu Z, Lin X, Wang S, Wen Y, Wang L (2024). Tumor microenvironment and immunotherapy for triple-negative breast cancer. Biomarker research.

[13] Eliassen AH, Missmer SA, Tworoger SS, Spiegelman D, Barbieri RL, Dowsett M (2006). Endogenous steroid hormone concentrations and risk of breast cancer among premenopausal women. Journal of the National Cancer Institute.

[14] Key T, Appleby P, Barnes I, Reeves G (2002). Endogenous sex hormones and breast cancer in postmenopausal women: reanalysis of nine prospective studies. Journal of the National Cancer Institute.

[15] Menarche menopause, breast cancer risk (2012). individual participant meta-analysis, including 118 964 women with breast cancer from 117 epidemiological studies. The Lancet Oncology.

[16] Ma H, Bernstein L, Pike MC, Ursin G (2006). Reproductive factors and breast cancer risk according to joint estrogen and progesterone receptor status: a meta-analysis of epidemiological studies. Breast cancer research: BCR.

[17] Guo Z, Zhu Z, Luo M, Cao Y, Lin X, Wu Q (2025). Efficacy of cyclin-dependent kinase inhibitors with concurrent proton pump inhibitors in patients with breast cancer: a systematic review and meta-analysis. The oncologist.

[18] Beaber EF, Buist DS, Barlow WE, Malone KE, Reed SD, Li CI (2014). Recent oral contraceptive use by formulation and breast cancer risk among women 20 to 49 years of age. Cancer Res.

[19] Bakken K, Fournier A, Lund E, Waaseth M, Dumeaux V, Clavel-Chapelon F (2011). Menopausal hormone therapy and breast cancer risk: impact of different treatments. The European Prospective Investigation into Cancer and Nutrition. International journal of cancer.

[20] Vogel VG, Costantino JP, Wickerham DL, Cronin WM, Cecchini RS, Atkins JN (2010). Update of the National Surgical Adjuvant Breast and Bowel Project Study of Tamoxifen and Raloxifene (STAR) P-2 Trial: Preventing breast cancer. Cancer prevention research (Philadelphia, Pa).

[21] Hvidtfeldt UA, Lange T, Andersen I, Diderichsen F, Keiding N, Prescott E (2013). Educational differences in postmenopausal breast cancer-quantifying indirect effects through health behaviors, body mass index and reproductive patterns. PloS one.

[22] van Noord-Zaadstra BM, Looman CW, Alsbach H, Habbema JD, te Velde ER, Karbaat J (1991). Delaying childbearing: effect of age on fecundity and outcome of pregnancy. BMJ (Clinical research ed).

[23] Simmons RG, Jennings V (2020). Fertility awareness-based methods of family planning. Best Pract Res Clin Obstet Gynaecol.

[24] Goisis A, Håberg SE, Hanevik HI, Magnus MC, Kravdal Ø (2020). The demographics of assisted reproductive technology births in a Nordic country. Hum Reprod.

[25] Zreik TG, Mazloom A, Chen Y, Vannucci M, Pinnix CC, Fulton S (2010). Fertility drugs and the risk of breast cancer: a meta-analysis and review. Breast Cancer Res Treat.

[26] Kohler BA, Sherman RL, Howlader N, Jemal A, Ryerson AB, Henry KA (2015). Annual Report to the Nation on the Status of Cancer, 1975-2011, Featuring Incidence of Breast Cancer Subtypes by Race/Ethnicity, Poverty, and State. Journal of the National Cancer Institute.

[27] Roque M, Haahr T, Geber S, Esteves SC, Humaidan P (2019). Fresh versus elective frozen embryo transfer in IVF/ICSI cycles: a systematic review and meta-analysis of reproductive outcomes. Hum Reprod Update.

[28] Fishel S (2018). First in vitro fertilization baby-this is how it happened. Fertility and sterility.

[29] Graham ME, Jelin A, Hoon AH Jr, Wilms Floet AM, Levey E, Graham EM (2023). Assisted reproductive technology: Short- and long-term outcomes. Dev Med Child Neurol.

[30] Ombelet W, Van Robays J (2015). Artificial insemination history: hurdles and milestones. Facts, views & vision in ObGyn.

[31] Kamel RM (2013). Assisted reproductive technology after the birth of louise brown. Journal of reproduction & infertility.

[32] Eskew AM, Jungheim ES (2017). A History of Developments to Improve in vitro Fertilization. Missouri medicine.

[33] Vassard D, Pinborg A, Kamper-Jorgensen M, Lyng Forman J, Glazer CH, Kroman N (2021). Assisted reproductive technology treatment and risk of breast cancer: a population-based cohort study. Hum Reprod.

[34] Howles CM, Alam V, Tredway D, Homburg R, Warne DW (2010). Factors related to successful ovulation induction in patients with WHO group II anovulatory infertility. Reproductive biomedicine online.

[35] Humaidan P, Papanikolaou EG, Kyrou D, Alsbjerg B, Polyzos NP, Devroey P (2012). The luteal phase after GnRH-agonist triggering of ovulation: present and future perspectives. Reproductive biomedicine online.

[36] Al-Fozan H, Al-Khadouri M, Tan SL, Tulandi T (2004). A randomized trial of letrozole versus clomiphene citrate in women undergoing superovulation. Fertility and sterility.

[37] Misso ML, Teede HJ, Hart R, Wong J, Rombauts L, Melder AM (2012). Status of clomiphene citrate and metformin for infertility in PCOS. Trends in endocrinology and metabolism: TEM.

[38] Kelly E (2003). Recombinant human follicle-stimulating hormone versus urinary-derived human menopausal gonadotropin for controlled ovarian stimulation: the science and art of assisted reproductive technologies. Fertility and sterility.

[39] Jayaprakasan K, Hopkisson J, Campbell B, Johnson I, Thornton J, Raine-Fenning N (2010). A randomised controlled trial of 300 versus 225 IU recombinant FSH for ovarian stimulation in predicted normal responders by antral follicle count. BJOG: an international journal of obstetrics and gynaecology.

[40] Filicori M, Fazleabas AT, Huhtaniemi I, Licht P, Rao Ch V, Tesarik J (2005). Novel concepts of human chorionic gonadotropin: reproductive system interactions and potential in the management of infertility. Fertility and sterility.

[41] Blumenfeld Z (2020). What Is the Best Regimen for Ovarian Stimulation of Poor Responders in ART/IVF?. Frontiers in endocrinology.

[42] Brinton LA (2012). Breast cancer risk after use of fertility drugs: stimulating new controversy. Journal of the National Cancer Institute.

[43] Labarta E, Rodríguez C (2020). Progesterone use in assisted reproductive technology. Best Pract Res Clin Obstet Gynaecol.

[44] Stewart LM, Holman CD, Hart R, Bulsara MK, Preen DB, Finn JC (2012). In vitro fertilization and breast cancer: is there cause for concern?. Fertility and sterility.

[45] Aydin B, Yoruk N (2023). Does in vitro fertilization affect the hearing levels of women?. European review for medical and pharmacological sciences.

[46] Bernstein L (2002). Epidemiology of endocrine-related risk factors for breast cancer. Journal of mammary gland biology and neoplasia.

[47] Labarta E, Mariani G, Holtmann N, Celada P, Remohí J, Bosch E (2017). Low serum progesterone on the day of embryo transfer is associated with a diminished ongoing pregnancy rate in oocyte donation cycles after artificial endometrial preparation: a prospective study. Hum Reprod.

[48] Klemetti R, Sevón T, Gissler M, Hemminki E (2005). Complications of IVF and ovulation induction. Hum Reprod.

[49] Carr BR, MacDonald PC, Simpson ER (1982). The role of lipoproteins in the regulation of progesterone secretion by the human corpus luteum. Fertility and sterility.

[50] Samavat H, Kurzer MS (2015). Estrogen metabolism and breast cancer. Cancer Lett.

[51] Yage J D, Davidson N E (2006). Estrogen Carcinogenesis in Breast Cancer. N Engl J Med.

[52] Raftogianis R, Creveling C, Weinshilboum R, Weisz J (2000). Estrogen metabolism by conjugation. Journal of the National Cancer Institute Monographs.

[53] Jefcoate CR, Liehr JG, Santen RJ, Sutter TR, Yager JD, Yue W (2000). Tissue-specific synthesis and oxidative metabolism of estrogens. Journal of the National Cancer Institute Monographs.

[54] Rogan EG, Badawi AF, Devanesan PD, Meza JL, Edney JA, West WW (2003). Relative imbalances in estrogen metabolism and conjugation in breast tissue of women with carcinoma: potential biomarkers of susceptibility to cancer. Carcinogenesis.

[55] Stanczyk FZ (2003). All progestins are not created equal. Steroids.

[56] Samson M, Porter N, Orekoya O, Hebert JR, Adams SA, Bennett CL (2016). Progestin and breast cancer risk: a systematic review. Breast Cancer Res Treat.

[57] Starek-Swiechowicz B, Budziszewska B, Starek A (2021). Endogenous estrogens-breast cancer and chemoprevention. Pharmacol Rep.

[58] Dent R, Trudeau M, Pritchard KI, Hanna WM, Kahn HK, Sawka CA (2007). Triple-negative breast cancer: clinical features and patterns of recurrence. Clinical cancer research: an official journal of the American Association for Cancer Research.

[59] Colditz GA (1998). Relationship between estrogen levels, use of hormone replacement therapy, and breast cancer. Journal of the National Cancer Institute.

[60] Calle EE, Feigelson HS, Hildebrand JS, Teras LR, Thun MJ, Rodriguez C (2009). Postmenopausal hormone use and breast cancer associations differ by hormone regimen and histologic subtype. Cancer.

[61] Trabert B, Sherman ME, Kannan N, Stanczyk FZ (2020). Progesterone and Breast Cancer. Endocr Rev.

[62] Pike MC, Spicer DV, Dahmoush L, Press MF (1993). Estrogens, progestogens, normal breast cell proliferation, and breast cancer risk. Epidemiologic reviews.

[63] Loboda A, Nebozhyn M, Klinghoffer R, Frazier J, Chastain M, Arthur W (2010). A gene expression signature of RAS pathway dependence predicts response to PI3K and RAS pathway inhibitors and expands the population of RAS pathway activated tumors. BMC medical genomics.

[64] Levin ER (2002). Cellular functions of plasma membrane estrogen receptors. Steroids.

[65] Govind AP, Thampan RV (2003). Membrane associated estrogen receptors and related proteins: localization at the plasma membrane and the endoplasmic reticulum. Molecular and cellular biochemistry.

[66] Bado I, Nikolos F, Rajapaksa G, Wu W, Castaneda J, Krishnamurthy S (2017). Somatic loss of estrogen receptor beta and p53 synergize to induce breast tumorigenesis. Breast cancer research: BCR.

[67] Lu W, Katzenellenbogen BS (2017). Estrogen Receptor-β Modulation of the ERα-p53 Loop Regulating Gene Expression, Proliferation, and Apoptosis in Breast Cancer. Hormones & cancer.

[68] Pardo I, Lillemoe HA, Blosser RJ, Choi M, Sauder CA, Doxey DK (2014). Next-generation transcriptome sequencing of the premenopausal breast epithelium using specimens from a normal human breast tissue bank. Breast cancer research: BCR.

[69] Graham JD, Mote PA, Salagame U, van Dijk JH, Balleine RL, Huschtscha LI (2009). DNA replication licensing and progenitor numbers are increased by progesterone in normal human breast. Endocrinology.

[70] Chen W, Wei W, Yu L, Ye Z, Huang F, Zhang L (2021). Mammary Development and Breast Cancer: a Notch Perspective. Journal of mammary gland biology and neoplasia.

[71] Brisken C, Park S, Vass T, Lydon JP, O'Malley BW, Weinberg RA (1998). A paracrine role for the epithelial progesterone receptor in mammary gland development. Proceedings of the National Academy of Sciences of the United States of America.

[72] Anderson E (2002). The role of oestrogen and progesterone receptors in human mammary development and tumorigenesis. Breast cancer research: BCR.

[73] Grimm SL, Hartig SM, Edwards DP (2016). Progesterone Receptor Signaling Mechanisms. Journal of molecular biology.

[74] Lange CA, Gioeli D, Hammes SR, Marker PC (2007). Integration of rapid signaling events with steroid hormone receptor action in breast and prostate cancer. Annual review of physiology.

[75] Mulac-Jericevic B, Conneely OM (2004). Reproductive tissue selective actions of progesterone receptors. Reproduction (Cambridge, England).

[76] Mote PA, Bartow S, Tran N, Clarke CL (2002). Loss of co-ordinate expression of progesterone receptors A and B is an early event in breast carcinogenesis. Breast Cancer Res Treat.

[77] Slepicka PF, Cyrill SL, Dos Santos CO (2019). Pregnancy and Breast Cancer: Pathways to Understand Risk and Prevention. Trends Mol Med.

[78] Howard BA, Gusterson BA (2000). Human breast development. Journal of mammary gland biology and neoplasia.

[79] Hewitt SC, Harrell JC, Korach KS (2005). Lessons in estrogen biology from knockout and transgenic animals. Annual review of physiology.

[80] Conneely OM, Mulac-Jericevic B, Lydon JP, De Mayo FJ (2001). Reproductive functions of the progesterone receptor isoforms: lessons from knock-out mice. Molecular and cellular endocrinology.

[81] Figueroa JD, Pfeiffer RM, Brinton LA, Palakal MM, Degnim AC, Radisky D (2016). Standardized measures of lobular involution and subsequent breast cancer risk among women with benign breast disease: a nested case-control study. Breast Cancer Res Treat.

[82] Figueroa JD, Pfeiffer RM, Patel DA, Linville L, Brinton LA, Gierach GL (2014). Terminal duct lobular unit involution of the normal breast: implications for breast cancer etiology. Journal of the National Cancer Institute.

[83] Schedin P, Mitrenga T, McDaniel S, Kaeck M (2004). Mammary ECM composition and function are altered by reproductive state. Molecular carcinogenesis.

[84] Reinier KS, Vacek PM, Geller BM (2007). Risk factors for breast carcinoma in situ versus invasive breast cancer in a prospective study of pre- and post-menopausal women. Breast Cancer Res Treat.

[85] Sainz B Jr, Carron E, Vallespinós M, Machado HL (2016). Cancer Stem Cells and Macrophages: Implications in Tumor Biology and Therapeutic Strategies. Mediators of inflammation.

[86] Elder AM, Tamburini BAJ, Crump LS, Black SA, Wessells VM, Schedin PJ (2018). Semaphorin 7A Promotes Macrophage-Mediated Lymphatic Remodeling during Postpartum Mammary Gland Involution and in Breast Cancer. Cancer Res.

[87] Atabai K, Sheppard D, Werb Z (2007). Roles of the innate immune system in mammary gland remodeling during involution. Journal of mammary gland biology and neoplasia.

[88] Montalto FI, De Amicis F (2020). Cyclin D1 in Cancer: A Molecular Connection for Cell Cycle Control, Adhesion and Invasion in Tumor and Stroma. Cells.

[89] De Amicis F, Chiodo C, Morelli C, Casaburi I, Marsico S, Bruno R (2019). AIB1 sequestration by androgen receptor inhibits estrogen-dependent cyclin D1 expression in breast cancer cells. BMC cancer.

[90] Beleut M, Rajaram RD, Caikovski M, Ayyanan A, Germano D, Choi Y (2010). Two distinct mechanisms underlie progesterone-induced proliferation in the mammary gland. Proceedings of the National Academy of Sciences of the United States of America.

[91] Fernandez-Valdivia R, Mukherjee A, Creighton CJ, Buser AC, DeMayo FJ, Edwards DP (2008). Transcriptional response of the murine mammary gland to acute progesterone exposure. Endocrinology.

[92] Potten CS, Watson RJ, Williams GT, Tickle S, Roberts SA, Harris M (1988). The effect of age and menstrual cycle upon proliferative activity of the normal human breast. British journal of cancer.

[93] Hilton HN, Santucci N, Silvestri A, Kantimm S, Huschtscha LI, Graham JD (2014). Progesterone stimulates progenitor cells in normal human breast and breast cancer cells. Breast Cancer Res Treat.

[94] Kannan N, Huda N, Tu L, Droumeva R, Aubert G, Chavez E (2013). The luminal progenitor compartment of the normal human mammary gland constitutes a unique site of telomere dysfunction. Stem cell reports.

[95] Tanaka H, Abe S, Huda N, Tu L, Beam MJ, Grimes B (2012). Telomere fusions in early human breast carcinoma. Proceedings of the National Academy of Sciences of the United States of America.

[96] Okoh VO, Felty Q, Parkash J, Poppiti R, Roy D (2013). Reactive oxygen species via redox signaling to PI3K/AKT pathway contribute to the malignant growth of 4-hydroxy estradiol-transformed mammary epithelial cells. PloS one.

[97] Anstead GM, Carlson KE, Katzenellenbogen JA (1997). The estradiol pharmacophore: ligand structure-estrogen receptor binding affinity relationships and a model for the receptor binding site. Steroids.

[98] Chang M (2011). Dual roles of estrogen metabolism in mammary carcinogenesis. BMB reports.

[99] Seeger H, Wallwiener D, Kraemer E, Mueck AO (2006). Comparison of possible carcinogenic estradiol metabolites: effects on proliferation, apoptosis and metastasis of human breast cancer cells. Maturitas.

[100] Lareef MH, Garber J, Russo PA, Russo IH, Heulings R, Russo J (2005). The estrogen antagonist ICI-182-780 does not inhibit the transformation phenotypes induced by 17-beta-estradiol and 4-OH estradiol in human breast epithelial cells. International journal of oncology.

[101] Santen R, Cavalieri E, Rogan E, Russo J, Guttenplan J, Ingle J (2009). Estrogen mediation of breast tumor formation involves estrogen receptor-dependent, as well as independent, genotoxic effects. Annals of the New York Academy of Sciences.

[102] Santen RJ, Yue W, Wang JP (2015). Estrogen metabolites and breast cancer. Steroids.

[103] Zahid M, Kohli E, Saeed M, Rogan E, Cavalieri E (2006). The greater reactivity of estradiol-3,4-quinone vs estradiol-2,3-quinone with DNA in the formation of depurinating adducts: implications for tumor-initiating activity. Chemical research in toxicology.

[104] Fussell KC, Udasin RG, Smith PJ, Gallo MA, Laskin JD (2011). Catechol metabolites of endogenous estrogens induce redox cycling and generate reactive oxygen species in breast epithelial cells. Carcinogenesis.

[105] Braz MG, Salvadori DM (2007). Lack of genotoxicity induced by endogenous and synthetic female sex hormones in peripheral blood cells detected by alkaline comet assay. Environmental and molecular mutagenesis.

[106] Aisemberg J, Vercelli CA, Bariani MV, Billi SC, Wolfson ML, Franchi AM (2013). Progesterone is essential for protecting against LPS-induced pregnancy loss. LIF as a potential mediator of the anti-inflammatory effect of progesterone. PloS one.

[107] Feser W, Kerdar RS, Baumann A, Körber J, Blode H, Kuhnz W (1998). DNA adduct formation of selected sex steroids in human liver slices in vitro. Toxicology in vitro: an international journal published in association with BIBRA.

[108] Gao N, Nester RA, Sarkar MA (2004). 4-Hydroxy estradiol but not 2-hydroxy estradiol induces expression of hypoxia-inducible factor 1alpha and vascular endothelial growth factor A through phosphatidylinositol 3-kinase/Akt/FRAP pathway in OVCAR-3 and A2780-CP70 human ovarian carcinoma cells. Toxicology and applied pharmacology.

[109] Ratcliffe PJ, O'Rourke JF, Maxwell PH, Pugh CW (1998). Oxygen sensing, hypoxia-inducible factor-1 and the regulation of mammalian gene expression. The Journal of experimental biology.

[110] Kwon YJ, Cho NH, Ye DJ, Baek HS, Ryu YS, Chun YJ (2018). Cytochrome P450 1B1 promotes cancer cell survival via specificity protein 1 (Sp1)-mediated suppression of death receptor 4. Journal of toxicology and environmental health Part A.

[111] Kwon YJ, Baek HS, Ye DJ, Shin S, Kim D, Chun YJ (2016). CYP1B1 Enhances Cell Proliferation and Metastasis through Induction of EMT and Activation of Wnt/β-Catenin Signaling via Sp1 Upregulation. PloS one.

[112] Chen ZH, Na HK, Hurh YJ, Surh YJ (2005). 4-Hydroxyestradiol induces oxidative stress and apoptosis in human mammary epithelial cells: possible protection by NF-kappaB and ERK/MAPK. Toxicology and applied pharmacology.

[113] Khongthong P, Roseweir AK, Edwards J (2019). The NF-KB pathway and endocrine therapy resistance in breast cancer. Endocrine-related cancer.

[114] Osborne MP, Bradlow HL, Wong GY, Telang NT (1993). Upregulation of estradiol C16 alpha-hydroxylation in human breast tissue: a potential biomarker of breast cancer risk. Journal of the National Cancer Institute.

[115] Suto A, Bradlow HL, Wong GY, Osborne MP, Telang NT (1993). Experimental down-regulation of intermediate biomarkers of carcinogenesis in mouse mammary epithelial cells. Breast Cancer Res Treat.

[116] Lee HJ, Ormandy CJ (2012). Interplay between progesterone and prolactin in mammary development and implications for breast cancer. Molecular and cellular endocrinology.

[117] Vonderhaar BK (1999). Prolactin involvement in breast cancer. Endocrine-related cancer.

[118] Wang M, Wu X, Chai F, Zhang Y, Jiang J (2016). Plasma prolactin and breast cancer risk: a meta- analysis. Scientific reports.

[119] Horseman ND, Gregerson KA (2014). Prolactin actions. Journal of molecular endocrinology.

[120] Gehring C, Siepmann T, Heidegger H, Jeschke U (2016). The controversial role of human chorionic gonadotropin in the development of breast cancer and other types of tumors. Breast (Edinburgh, Scotland).

[121] Rahman NA, Rao CV (2009). Recent progress in luteinizing hormone/human chorionic gonadotrophin hormone research. Molecular human reproduction.

[122] Schuler-Toprak S, Treeck O, Ortmann O (2017). Human Chorionic Gonadotropin and Breast Cancer. Int J Mol Sci.

[123] Brinton LA, Scoccia B, Moghissi KS, Westhoff CL, Niwa S, Ruggieri D (2014). Long-term relationship of ovulation-stimulating drugs to breast cancer risk. Cancer epidemiology, biomarkers & prevention: a publication of the American Association for Cancer Research, cosponsored by the American Society of Preventive Oncology.

[124] Moher D, Liberati A, Tetzlaff J, Altman DG (2010). Preferred reporting items for systematic reviews and meta-analyses: the PRISMA statement. International journal of surgery (London, England).

[125] Pappo I, Lerner-Geva L, Halevy A, Olmer L, Friedler S, Raziel A (2008). The possible association between IVF and breast cancer incidence. Annals of surgical oncology.

[126] Froehlich K, Schmidt A, Heger JI, Al-Kawlani B, Aberl CA, Jeschke U (2019). Breast cancer, placenta and pregnancy. Eur J Cancer.

[127] Hanf V, Hanf D (2014). Reproduction and breast cancer risk. Breast Care (Basel).

[128] Gauthier E, Paoletti X, Clavel-Chapelon F (2004). Breast cancer risk associated with being treated for infertility: results from the French E3N cohort study. Hum Reprod.

[129] Tsafrir A, Lerner-Geva L, Zaslavsky-Paltiel I, Laufer N, Simon A, Einav S (2020). Cancer in IVF patients treated at age 40 years and older: long term follow-up. Reproductive biomedicine online.

[130] Reigstad MM, Larsen IK, Myklebust T, Robsahm TE, Oldereid NB, Omland AK (2015). Risk of breast cancer following fertility treatment-a registry based cohort study of parous women in Norway. International journal of cancer.

[131] Lerner-Geva L, Rabinovici J, Olmer L, Blumstein T, Mashiach S, Lunenfeld B (2012). Are infertility treatments a potential risk factor for cancer development? Perspective of 30 years of follow-up. Gynecological endocrinology: the official journal of the International Society of Gynecological Endocrinology.

[132] Silva Idos S, Wark PA, McCormack VA, Mayer D, Overton C, Little V (2009). Ovulation-stimulation drugs and cancer risks: a long-term follow-up of a British cohort. British journal of cancer.

[133] Calderon-Margalit R, Friedlander Y, Yanetz R, Kleinhaus K, Perrin MC, Manor O (2009). Cancer risk after exposure to treatments for ovulation induction. Am J Epidemiol.

[134] Kristiansson P, Björ O, Wramsby H (2007). Tumour incidence in Swedish women who gave birth following IVF treatment. Hum Reprod.

[135] Lerner-Geva L, Keinan-Boker L, Blumstein T, Boyko V, Olmar L, Mashiach S (2006). Infertility, ovulation induction treatments and the incidence of breast cancer-a historical prospective cohort of Israeli women. Breast Cancer Res Treat.

[136] Brinton LA, Scoccia B, Moghissi KS, Westhoff CL, Althuis MD, Mabie JE (2004). Breast cancer risk associated with ovulation-stimulating drugs. Hum Reprod.

[137] Burkman RT, Tang MT, Malone KE, Marchbanks PA, McDonald JA, Folger SG (2003). Infertility drugs and the risk of breast cancer: findings from the National Institute of Child Health and Human Development Women's Contraceptive and Reproductive Experiences Study. Fertility and sterility.

[138] Reigstad MM, Storeng R, Myklebust TA, Oldereid NB, Omland AK, Robsahm TE (2017). Cancer Risk in Women Treated with Fertility Drugs According to Parity Status-A Registry-based Cohort Study. Cancer epidemiology, biomarkers & prevention: a publication of the American Association for Cancer Research, cosponsored by the American Society of Preventive Oncology.

[139] Williams CL, Jones ME, Swerdlow AJ, Botting BJ, Davies MC, Jacobs I (2018). Risks of ovarian, breast, and corpus uteri cancer in women treated with assisted reproductive technology in Great Britain, 1991-2010: data linkage study including 2.2 million person years of observation. BMJ (Clinical research ed).

[140] Lundberg FE, Johansson AL, Rodriguez-Wallberg K, Brand JS, Czene K, Hall P (2016). Association of infertility and fertility treatment with mammographic density in a large screening-based cohort of women: a cross-sectional study. Breast cancer research: BCR.

[141] Lundberg FE, Iliadou AN, Rodriguez-Wallberg K, Bergh C, Gemzell-Danielsson K, Johansson ALV (2017). Ovarian stimulation and risk of breast cancer in Swedish women. Fertility and sterility.

[142] Luke B, Brown MB, Spector LG, Missmer SA, Leach RE, Williams M (2015). Cancer in women after assisted reproductive technology. Fertility and sterility.

[143] Brinton LA, Trabert B, Shalev V, Lunenfeld E, Sella T, Chodick G (2013). In vitro fertilization and risk of breast and gynecologic cancers: a retrospective cohort study within the Israeli Maccabi Healthcare Services. Fertility and sterility.

[144] Fei C, Deroo LA, Sandler DP, Weinberg CR (2012). Fertility drugs and young-onset breast cancer: results from the Two Sister Study. Journal of the National Cancer Institute.

[145] Källén B, Finnström O, Lindam A, Nilsson E, Nygren KG, Olausson PO (2011). Malignancies among women who gave birth after in vitro fertilization. Hum Reprod.

[146] Derks-Smeets IAP, Schrijver LH, de Die-Smulders CEM, Tjan-Heijnen VCG, van Golde RJT, Smits LJ (2018). Ovarian stimulation for IVF and risk of primary breast cancer in BRCA1/2 mutation carriers. British journal of cancer.

[147] Perri T, Naor-Revel S, Eliassi-Revivo P, Lifshitz D, Friedman E, Korach J (2021). Fertility treatments and breast cancer risk in Jewish Israeli BRCA mutation carriers. Fertility and sterility.

[148] Machtinger R, Fallach N, Goldstein I, Chodick G, Schiff E, Orvieto R (2022). Ovarian stimulation for fertility treatments and risk of breast cancer: a matched cohort study. Hum Reprod.

[149] Taheripanah R, Balash F, Anbiaee R, Mahmoodi M, Akbari Sene A (2018). Breast Cancer and Ovulation Induction Treatments. Clinical breast cancer.

[150] van den Belt-Dusebout AW, Spaan M, Lambalk CB, Kortman M, Laven JS, van Santbrink EJ (2016). Ovarian Stimulation for In Vitro Fertilization and Long-term Risk of Breast Cancer. Jama.

[151] Kessous R, Davidson E, Meirovitz M, Sergienko R, Sheiner E (2016). The risk of female malignancies after fertility treatments: a cohort study with 25-year follow-up. Journal of cancer research and clinical oncology.

[152] Yli-Kuha AN, Gissler M, Klemetti R, Luoto R, Hemminki E (2012). Cancer morbidity in a cohort of 9175 Finnish women treated for infertility. Hum Reprod.

[153] Orgéas CC, Sanner K, Hall P, Conner P, Holte J, Nilsson SJ (2009). Breast cancer incidence after hormonal infertility treatment in Sweden: a cohort study. American journal of obstetrics and gynecology.

[154] Jensen A, Sharif H, Svare EI, Frederiksen K, Kjaer SK (2007). Risk of breast cancer after exposure to fertility drugs: results from a large Danish cohort study. Cancer epidemiology, biomarkers & prevention: a publication of the American Association for Cancer Research, cosponsored by the American Society of Preventive Oncology.

[155] Lerner-Geva L The possible association between in vitro fertilization treatments and caner development. 2003; 13: 23-27.

[156] Cetin I, Cozzi V, Antonazzo P (2008). Infertility as a cancer risk factor - a review. Placenta.

[157] Turan V, Lambertini M, Lee DY, Wang E, Clatot F, Karlan BY (2021). Association of Germline BRCA Pathogenic Variants With Diminished Ovarian Reserve: A Meta-Analysis of Individual Patient-Level Data. Journal of clinical oncology: official journal of the American Society of Clinical Oncology.

[158] Daum H, Peretz T, Laufer N (2018). BRCA mutations and reproduction. Fertility and sterility.

[159] Liu X, Yue J, Pervaiz R, Zhang H, Wang L (2022). Association between fertility treatments and breast cancer risk in women with a family history or BRCA mutations: a systematic review and meta-analysis. Frontiers in endocrinology.

[160] Tomao F, Lo Russo G, Spinelli GP, Tomao S (2014). Clinical use of fertility agents and risk of breast cancer: a recent update for an old problem. Curr Opin Obstet Gynecol.

[161] Momenimovahed Z, Taheri S, Tiznobaik A, Salehiniya H (2019). Do the Fertility Drugs Increase the Risk of Cancer? A Review Study. Frontiers in endocrinology.

[162] Sergentanis TN, Diamantaras AA, Perlepe C, Kanavidis P, Skalkidou A, Petridou ET (2014). IVF and breast cancer: a systematic review and meta-analysis. Hum Reprod Update.

[163] Li LL, Zhou J, Qian XJ, Chen YD (2013). Meta-analysis on the possible association between in vitro fertilization and cancer risk. International journal of gynecological cancer: official journal of the International Gynecological Cancer Society.

[164] Cullinane C, Gillan H, Geraghty J, Evoy D, Rothwell J, McCartan D (2022). Fertility treatment and breast-cancer incidence: meta-analysis. BJS open.

[165] Miller WBJVYoPR Differences between fertility desires and intentions: Implications for theory, research and policy. 2011: 75-98.

[166] Giaquinto AN, Sung H, Miller KD, Kramer JL, Newman LA, Minihan A (2022). Breast Cancer Statistics, 2022. CA: a cancer journal for clinicians.

[167] Nordeng H, Ystrøm E, Einarson A (2010). Perception of risk regarding the use of medications and other exposures during pregnancy. European journal of clinical pharmacology.

[168] Diezi AS, Vanetti M, Robert M, Schaad B, Baud D, Horsch A (2023). Informing about childbirth without increasing anxiety: a qualitative study of first-time pregnant women and partners' perceptions and needs. BMC pregnancy and childbirth.

[169] Breslow NE, Day NE (1987). Statistical methods in cancer research. Volume II-The design and analysis of cohort studies. IARC scientific publications.

[170] Russo J, Balogh GA, Russo IH (2008). Full-term pregnancy induces a specific genomic signature in the human breast. Cancer epidemiology, biomarkers & prevention: a publication of the American Association for Cancer Research, cosponsored by the American Society of Preventive Oncology.

[171] Miller N, Delbecchi L, Petitclerc D, Wagner GF, Talbot BG, Lacasse P (2006). Effect of stage of lactation and parity on mammary gland cell renewal. Journal of dairy science.

[172] Dos Santos CO, Dolzhenko E, Hodges E, Smith AD, Hannon GJ (2015). An epigenetic memory of pregnancy in the mouse mammary gland. Cell reports.

[173] Nynca A, Swigonska S, Molcan T, Petroff BK, Ciereszko RE (2023). Molecular Action of Tamoxifen in the Ovaries of Rats with Mammary Neoplasia. Int J Mol Sci.

[174] Choudhury S, Almendro V, Merino VF, Wu Z, Maruyama R, Su Y (2013). Molecular profiling of human mammary gland links breast cancer risk to a p27(+) cell population with progenitor characteristics. Cell stem cell.

[175] Taouk L, Gunthert K, Schulkin J (2023). Risk perception in pregnancy: Patient-physician discrepancies, information consumption, and mental health outcomes. Birth (Berkeley, Calif).

[176] His M, Gunter MJ, Keski-Rahkonen P, Rinaldi S Application of Metabolomics to Epidemiologic Studies of Breast Cancer: New Perspectives for Etiology and Prevention. Journal of clinical oncology: official journal of the American Society of Clinical Oncology. 2023: Jco2202754.

[177] [Breast cancer treatment guidelines(2022 edition)] Zhonghua zhong liu za zhi [Chinese journal of oncology]. 2023; 45: 803-33.

[178] Honma N, Yoshida M, Kinowaki K, Horii R, Katsurada Y, Murata Y (2023). The Japanese breast cancer society clinical practice guidelines for pathological diagnosis of breast cancer, 2022 edition. Breast cancer (Tokyo, Japan).

[179] Gradishar WJ, Moran MS, Abraham J, Aft R, Agnese D, Allison KH (2022). Breast cancer, version 3.2022, NCCN clinical practice guidelines in oncology. J Natl Compr Canc Netw.

[180] Song X, Chu J, Guo Z, Wei Q, Wang Q, Hu W (2024). Prognostic prediction of breast cancer patients using machine learning models: a retrospective analysis. Gland surgery.

[181] Loibl S, Poortmans P, Morrow M, Denkert C, Curigliano G (2021). Breast cancer. Lancet.

[182] Wang S, Wu W, Lin X, Zhang KM, Wu Q, Luo M (2023). Predictive and prognostic biomarkers of bone metastasis in breast cancer: current status and future directions. Cell Biosci.

[183] Blackwood O, Deb R (2020). Multidisciplinary team approach in breast cancer care: Benefits and challenges. Indian journal of pathology & microbiology.

[184] Maas P, Barrdahl M, Joshi AD, Auer PL, Gaudet MM, Milne RL (2016). Breast Cancer Risk From Modifiable and Nonmodifiable Risk Factors Among White Women in the United States. JAMA oncology.

[185] Lee A, Mavaddat N, Wilcox AN, Cunningham AP, Carver T, Hartley S (2019). BOADICEA: a comprehensive breast cancer risk prediction model incorporating genetic and nongenetic risk factors. Genetics in medicine: official journal of the American College of Medical Genetics.

[186] Pesapane F, Battaglia O, Pellegrino G, Mangione E, Petitto S, Fiol Manna ED (2023). Advances in breast cancer risk modeling: integrating clinics, imaging, pathology and artificial intelligence for personalized risk assessment. Future oncology (London, England).

