# Supplementary material for: Conception and Concern: A Review of Breast Cancer Risk in Assisted Reproductive Technology
Source: Int J Biol Sci. 2025 Mar 24;21(6):2647–71. doi: 10.7150/ijbs.105357 (PMC12035894; doi:10.7150/ijbs.105357)

Table S1: Possible research errors factors

Descriptions of the issue			Codes	Remarks
Problems in experimental design	A relatively small sample size or poor representation of selected study populations	Cohort	A	Calculation of sample size needed for a cohort study: $N = (Z\alpha*(2P*(1-P)^{(-1)} + Z\beta*(P1(1-P1) + P0*(1-P0))^{(-1)})^2 / (P1 - P0)^2$.<br>Typically, $Z\alpha=1.96$ $Z\beta=1.282$ ($\alpha=0.05$, $\beta=0.1$). P1 and P0 represent the incidence of disease or exposure in the case and control groups, respectively, where $P1=P0*RR$. The final N is usually increased by 10% to accommodate lost visits.
		Case-Control	B	Calculation of sample size needed for a Case-Control study: $N= [(Z\alpha/2 + Z\beta)^2 \times (P1 \times (1-P1) + P2 \times (1-P2))] / (P1 - P2)^2$.<br>Typically, $Z\alpha=1.96$ $Z\beta=1.282$ ($\alpha=0.05$, $\beta=0.1$). P1 and P2 represent the incidence of exposure in the case and control groups, respectively. The final N is typically expanded by 10% to account for missed visits.
	Imprecise clinical data of the patients enrolled	Incomplete description of the study object itself	C	Specific etiology of infertility (subgroup analyses showed that women with non-ovulatory causes had a higher risk of breast cancer - supporting the idea that fertility drugs and the reason for the treatment are two independent risk factors for breast cancer), type of ART treatment, dosage, duration of exposure to the drug, and number of cycles of the treatment, and the outcome of the ART treatment are not clearly stated.
		Lacked control over interference caused by non-research factors	D	Includes confounding factors such as age of study participants, reproductive history (parity, age at first birth, etc.), use of contraceptives or HRT, family history of breast cancer, and other confounding factors that are not related to the study factors but can affect the risk of developing breast cancer.<br>Non-experimental factors were completely omitted in some studies, while in others, non-experimental factors could not be controlled for due to differences in the conditions of the experimental and control groups (e.g., the average level of educational attainment was higher in the ART group than in the control group).
Problems in the experimental implementation	Short duration of follow-up years (mean time less than 10 years significantly or follow-up ends well before the age of high breast cancer incidence)		E	Some studies have found a higher risk of breast cancer after more than 10 years of follow-up, and false-negative results may occur when the follow-up period is too short.

	A very small number of breast cancer cases in the follow-up	F	Since the incidence of breast cancer is relatively low (approximately 0.03% in the population), it is common to encounter in cohort studies that the sample size of patients in the ART treatment group who ultimately develop breast cancer is too small. This may lead to confounding by irrelevant factors, resulting in decreased credibility of the experimental results.
	Loss of study participants or some unavailable key information	G	The loss of study participants, particularly breast cancer patients, has an impact on the results. Furthermore, certain details regarding the treatment process, including fluctuations in estrogen and progesterone levels throughout the treatment, and the outcome of achieving a successful full-term pregnancy after undergoing ART, are unavailable or missing.
Problems in experimental statistics	Inaccurate selected statistics	H	Many studies use the SIR as a statistical parameter, but often fail to consider other factors that influence breast cancer risk.

Figure S1: Pathways of steroid hormone synthesis

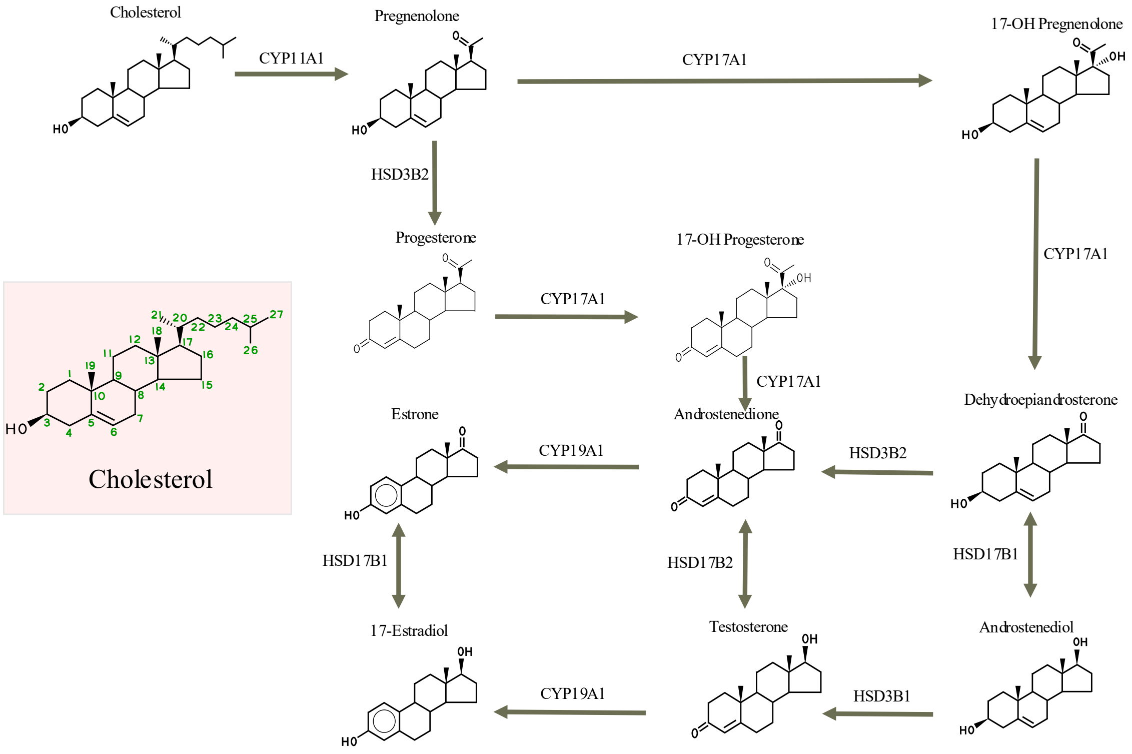

Figure S2: Endogenous estrogen metabolism

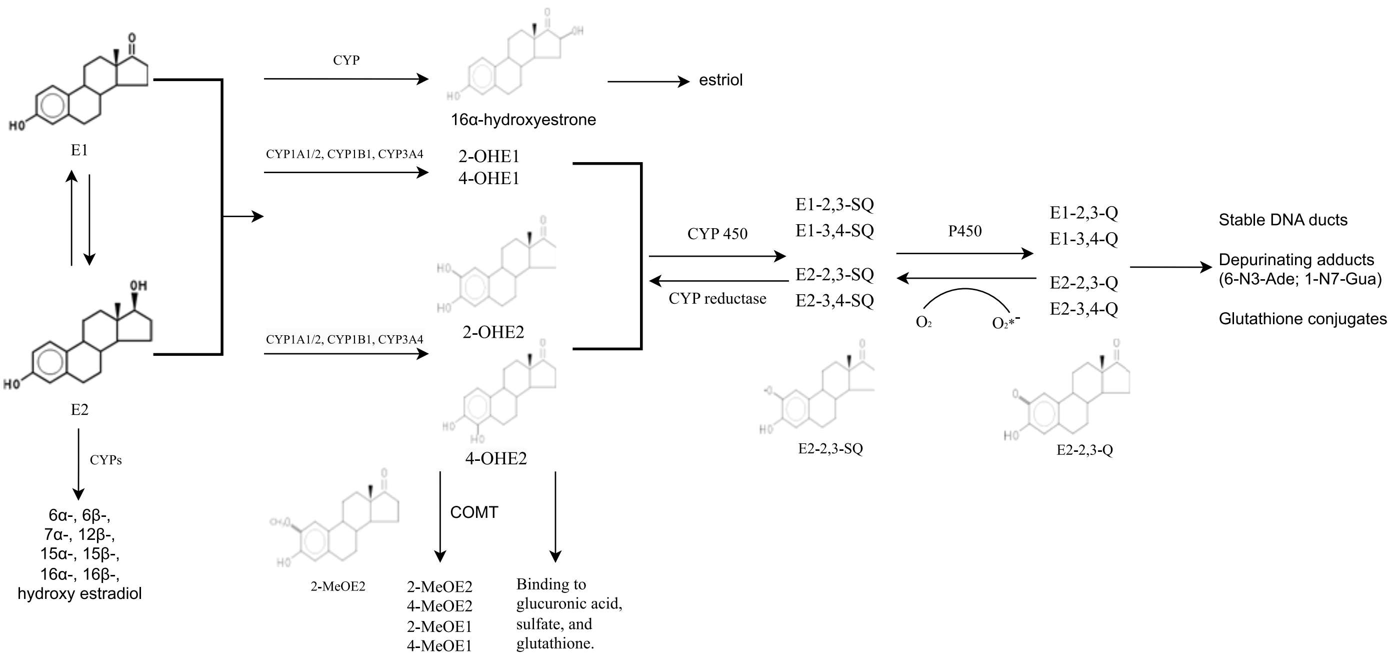

Supplement: Supplementary file 1 — Supplementary figures and table. [file ijbsv21p2647s1.pdf]
